# Lignin from hydrothermally pretreated grass biomass retards enzymatic cellulose degradation by acting as a physical barrier rather than by inducing nonproductive adsorption of enzymes

**DOI:** 10.1186/s13068-018-1085-0

**Published:** 2018-04-02

**Authors:** Demi T. Djajadi, Mads M. Jensen, Marlene Oliveira, Anders Jensen, Lisbeth G. Thygesen, Manuel Pinelo, Marianne Glasius, Henning Jørgensen, Anne S. Meyer

**Affiliations:** 1 Department of Chemical and Biochemical Engineering, Technical University of Denmark, Søltofts Plads Building 229, 2800 Kongens Lyngby, Denmark; 2 Department of Chemistry, Aarhus University, Langelandsgade 140, 8000 Aarhus C, Denmark; 3 Department of Geosciences and Natural Resource Management, University of Copenhagen, Rolighedsvej 23, 1958 Frederiksberg C, Denmark; 4 Present Address: Department of Plant and Environmental Sciences, University of Copenhagen, Thorvaldsensvej 40, 1871 Frederiksberg C, Denmark

**Keywords:** Lignin, Cellulases, Adsorption, Inhibition, Enzymatic hydrolysis, *S*/*G* ratio, β-*O*-4 linkage, Apparent surface abundance, Depolymerization, Physical barrier

## Abstract

**Background:**

Lignin is known to hinder efficient enzymatic conversion of lignocellulose in biorefining processes. In particular, nonproductive adsorption of cellulases onto lignin is considered a key mechanism to explain how lignin retards enzymatic cellulose conversion in extended reactions.

**Results:**

Lignin-rich residues (LRRs) were prepared via extensive enzymatic cellulose degradation of corn stover (*Zea mays* subsp. *mays* L.), *Miscanthus* ×* giganteus* stalks (MS) and wheat straw (*Triticum aestivum* L.) (WS) samples that each had been hydrothermally pretreated at three severity factors (log *R*_0_) of 3.65, 3.83 and 3.97. The LRRs had different residual carbohydrate levels—the highest in MS; the lowest in WS. The residual carbohydrate was not traceable at the surface of the LRRs particles by ATR-FTIR analysis. The chemical properties of the lignin in the LRRs varied across the three types of biomass, but monolignols composition was not affected by the severity factor. When pure cellulose was added to a mixture of LRRs and a commercial cellulolytic enzyme preparation, the rate and extent of glucose release were unaffected by the presence of LRRs regardless of biomass type and severity factor, despite adsorption of the enzymes to the LRRs. Since the surface of the LRRs particles were covered by lignin, the data suggest that the retardation of enzymatic cellulose degradation during extended reaction on lignocellulosic substrates is due to physical blockage of the access of enzymes to the cellulose caused by the gradual accumulation of lignin at the surface of the biomass particles rather than by nonproductive enzyme adsorption.

**Conclusions:**

The study suggests that lignin from hydrothermally pretreated grass biomass retards enzymatic cellulose degradation by acting as a physical barrier blocking the access of enzymes to cellulose rather than by inducing retardation through nonproductive adsorption of enzymes.

**Electronic supplementary material:**

The online version of this article (10.1186/s13068-018-1085-0) contains supplementary material, which is available to authorized users.

## Background

Optimal utilization of lignocellulosic biomass is vital for sustainable production of food, feed, fuels, chemicals, and materials. Hydrothermal pretreatment (HTP) and other types of physicochemical pretreatment methods are used to overcome the recalcitrance of the lignocellulosic biomass feedstocks to allow efficient enzymatic and biological processing [1, 2]. HTP of lignocellulosic biomass is known to remove parts of the hemicellulose fraction, thereby resulting in a cellulose-enriched fiber fraction which is more amenable to cellulase-catalyzed saccharification; depending on the pretreatment severity [3].

On the other hand, the presence of lignin in hydrothermally pretreated lignocellulosic biomass has also been considered as an important limiting factor in the enzymatic hydrolysis of cellulose [4, 5]. Lignin has thus been reported to promote nonproductive adsorption of the enzymes through charged and noncharged interactions and it may also act as a physical barrier that blocks the access of cellulolytic enzymes to cellulose [6–8]. Notably, nonproductive adsorption of cellulases to lignin has been considered as a key factor that limits the enzymatic conversion of pretreated biomass [4, 6, 9].

Available studies [10–18] have consistently shown that the enzymes are bound to isolated lignin materials from various biomasses and consequently the enzymes’ activity and/or the rate and extent of saccharification of model cellulose substrate in the presence of the isolated lignin were reduced. Observation of the latter has contributed to the use of the term “inhibitory effect” of lignin to enzymatic hydrolysis of cellulose in scientific literature.

Investigating the role of lignin as a physical barrier, however, can be difficult and complicated. Primarily this is due to modification of the lignin structure [19, 20] and its redistribution in the cell wall matrix [21, 22] after hydrothermal or dilute acid pretreatment. Advanced microscopy and imaging techniques have been developed to visualize components of lignocellulosic biomass, although extracting quantitative information can sometimes be difficult [23].

Recently, we have published a systematic study where industrially relevant Poaceae biomass feedstocks, namely corn stover, *Miscanthus *× *giganteus* stalks and wheat straw were hydrothermally pretreated at different severity levels. Via utilizing several quantitative and semiquantitative approaches, we proposed that surface properties, including apparent surface abundance of lignin as semiquantitatively determined by attenuated total reflectance Fourier transform infrared (ATR-FTIR) spectroscopy were correlated to the digestibility of biomass [24].

The objective of the present study was to further elucidate the role of lignin in retarding enzymatic cellulose degradation. This was done by simultaneously studying both enzyme–lignin interaction and the physicochemical properties and apparent surface abundance of lignocellulose components. The experimental approach was performed in three steps. Firstly, lignin-rich residues (LRRs) from the abovementioned biomass feedstocks were isolated through extensive enzymatic hydrolysis and characterized comprehensively. The ensuing profile of the LRRs is expected to resemble the actual bioprocess residues from lignocellulosic ethanol/biorefinery plants and the procedure is also expected to only exert minimal changes to the lignin structure. Secondly, the LRRs were assessed for their effect on the activity of state-of-the-art commercial cellulolytic enzyme mixture using relevant dosage. Thirdly, the data obtained in previous steps were extrapolated to explain what seemed to have occurred during actual extended enzymatic biomass hydrolysis reaction.

## Results and discussion

### Composition of lignin-rich residues

The composition of LRRs was assessed after repeated rounds of cellulase treatment at high enzyme loading followed by protease treatment to remove the enzymes adsorbed. In this way, the lignin isolation method also serves as exaggerated version of enzymatic cellulose degradation. As expected, the results revealed that the composition of the LRRs varied across biomass and pretreatment severities (Table 1). The LRRs from wheat straw (WS) had significantly higher lignin content than those from corn stover (CS) and *Miscanthus *× *giganteus* stalks (MS) at corresponding severities. In all LRRs from the three biomasses, it was observed that the lignin content increased relative to the carbohydrate content with elevated pretreatment severity (Table 1). The significantly higher residual carbohydrates content in the LRRs of MS is in agreement with our previous finding that MS was more recalcitrant compared to CS and WS [24] even though the starting compositions of the materials at corresponding pretreatment severities were similar (Table 1).

**Table 1:** Composition of pretreated biomass feedstocks and their resulting isolated lignin-rich residues

Biomass—log *R*_*0*_	Pretreated biomass feedstocks			Lignin-rich residues		
	Glucan	Xylan	Lignin^1^	Glucan	Xylan	Lignin^1^
	(% w/w DM)					
CS—3.65	55.5 ± 3.1^ab^	14.7 ± 0.8^a^	23.8 ± 2.3^cd^	20.9 ± 0.5^b^	5.4 ± 0.2^a^	60.4 ± 1.3^f^
CS—3.83	55.7 ± 1.3^ab^	11.2 ± 0.5^b^	22.4 ± 0.8^d^	14.5 ± 0.1^d^	3.2 ± 0.1^bc^	75.4 ± 0.9^de^
CS—3.97	61.2 ± 1.1^a^	6.4 ± 0.1^e^	19.9 ± 3.9^d^	7.2 ± 0.2^f^	1.6 ± 0.0^e^	79.5 ± 1.6^bc^
MS—3.65	53.6 ± 2.6^b^	11.3 ± 0.4^b^	32.5 ± 2.1^ab^	33.9 ± 0.7^a^	5.7 ± 0.1^a^	58.5 ± 0.7^f^
MS—3.83	54.7 ± 2.8^ab^	7.8 ± 0.6^d^	32.2 ± 0.5^ab^	18.9 ± 0.6^c^	3.0 ± 0.1^c^	73.1 ± 0.8^e^
MS—3.97	55.9 ± 2.1^ab^	4.5 ± 0.2^f^	35.6 ± 0.3^a^	11.7 ± 0.5^e^	1.4 ± 0.0^e^	81.9 ± 0.3^b^
WS—3.65	54.8 ± 0.6^ab^	14.7 ± 0.0^a^	29.3 ± 0.7^bc^	13.7 ± 0.6^d^	3.4 ± 0.1^b^	77.7 ± 0.7^cd^
WS—3.83	58.2 ± 4.7^ab^	9.8 ± 0.4^c^	30.8 ± 0.7^ab^	7.9 ± 0.0^f^	2.0 ± 0.0^d^	86.2 ± 0.1^a^
WS—3.97	61.2 ± 2.5^a^	6.5 ± 0.2^e^	30.3 ± 1.1^b^	5.3 ± 0.1^g^	1.1 ± 0.0^f^	87.8 ± 1.0^a^

Results are average and standard deviation of triplicate measurements

*CS* corn stover, *MS Miscanthus *× *giganteus* stalks, *WS* wheat straw

Different letters indicate significant statistical difference based on ANOVA (*P *≤ 0.05)

^1^Based on acid insoluble lignin (AIL) and acid soluble lignin (ASL) contents

In order to investigate the accessibility of the residual carbohydrates, further enzymatic hydrolysis was done on the LRRs. The results showed that only negligible amounts of monosaccharides were released (Additional file 1: Table S1). This indicated that the remaining cellulose and hemicellulose fractions were not accessible to the enzymes; most likely due to the surface coverage by lignin. Therefore it can be assumed that adsorption of the enzymes onto residual cellulose is negligible.

In order to assess the apparent surface abundance of lignocellulose components in the LRRs, ATR-FTIR spectroscopy was utilized as described previously [24]. However, when using the semiquantitative approach, the peak area values of the carbohydrates in the lignin-rich residues (LRRs) were too low for reasonable quantification. Upon examination of the ATR-FTIR spectra (Additional file 1: Figures S1–S3), apparently this was due to the diminishing intensity of carbohydrate peaks corresponding to cellulose (895 cm^−1^) and hemicellulose (1732 cm^−1^) after extensive enzymatic hydrolysis process. On the other hand, the peaks corresponding to lignin (835, 1419, 1432, 1508 and 1601 cm^−1^) increased greatly after hydrolysis. Since ATR-FTIR wavenumbers have limited depth of penetration (Table 3), these observations confirmed the previous observations and inference (Additional file 1: Table S1) that the carbohydrates in the lignin-rich residues were not present in the surface, conceivably due to being engulfed by lignin. A likely scenario therefore was that as cellulose hydrolysis progressed, the work of the enzymes was halted due to the increasing presence of lignin in the surface of the biomass particles which acted as physical barrier. However, since lignin has been reported to promote retardation through nonproductive adsorption, the interaction between lignin and LRRs should also be investigated.

### Interaction between enzymes and lignin-rich residues

**Fig. 1:**
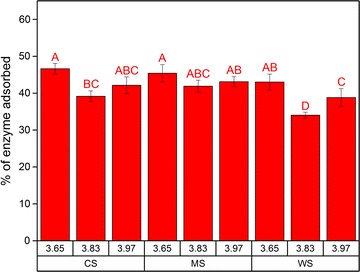
Adsorption of Cellic^®^ CTec3 with 20 mg protein/g DM loading on the lignin-rich residues (10 mg/ml) isolated from corn stover (CS), *Miscanthus *× *giganteus* stalks (MS), and wheat straw (WS) that were hydrothermally pretreated at different severity factors (log *R*_0_). Data points represent average and standard deviation from three experimental replicates. Different letters (A, B, C, …) indicate significant statistical difference based on ANOVA (*P* ≤ 0.05)

In order to directly assess the interaction between the LRRs from the pretreated biomass with a commercial cellulolytic enzyme mixture (Cellic^®^ CTec3), an adsorption experiment was performed. No consistent trends were evident across all LRRs regardless whether it was based on biomass feedstocks or severity factors. Approximately 34–47% of total protein in the enzyme mixture was adsorbed in all cases (Fig. 1).

Several studies on the adsorption of cellulases on lignin materials isolated from various biomasses have found that adsorption of cellulases (or their adsorption parameters modeled with Langmuir isotherms) increase slightly with elevated severity factor [15, 25, 26], although one study found the opposite [27]. In one study on lignin isolated from corn stover that had been hydrothermally pretreated at different severities, it was found that there were only minor differences in the binding capacity of cellulases, with only 6% of increase with severity factor (log *R*_0_) of 3.6–3.9 [26]. These results imply that the applied severity factor during pretreatment does not always warrant a significant increase in adsorption of cellulolytic enzymes. However, separate tests need to be conducted to assess whether adsorption of enzymes has direct impact on their performance.

**Fig. 2:**
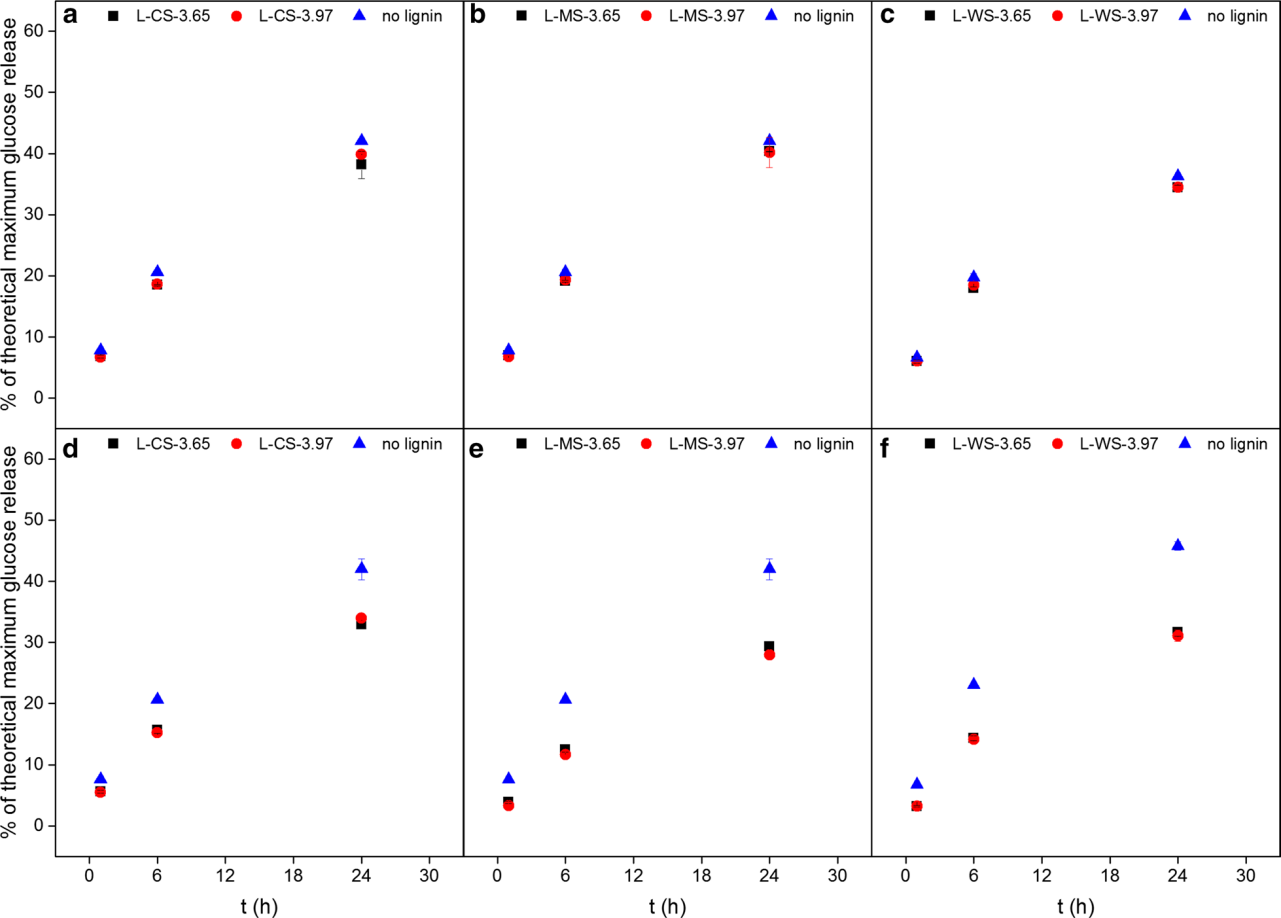
Glucose release from 2% DM Avicel hydrolysis after adsorption experiment of Cellic^®^ CTec3 in the presence of lignin-rich residues (LRRs) as in Experiment I (**a**–**c**) or by supernatant containing unbound enzymes after incubation with LRRs in Experiment II (**d**–Pyrolysis-GC–MS characterization of forage materials). LRRs were isolated from corn stover (L-CS) (**a** and **d**), *Miscanthus *× *giganteus* stalks (L-MS) (**b** and **e**), and wheat straw (L-WS) (**c** and **f**) that were hydrothermally pretreated at severity factors (log *R*_0_) 3.65 and 3.97. Data points represent average and standard deviation from two replicates

Therefore, in order to further study the interaction of the LRRs with cellulolytic enzymes, the effect of LRRs on activity of the enzymes was examined. The assessment was performed in two sets of experiments. In the first experiment (Experiment I), an Avicel (cellulose) suspension was added to the mixture of preincubated Cellic^®^ CTec3 and LRRs to directly assess both nonproductive adsorption and any consequent “inhibitory” effect of the LRRs. In the second experiment (Experiment II), each supernatant after preincubation of Cellic^®^ CTec3 and LRRs was added to an Avicel suspension to assess the significance of the adsorption of enzymes onto LRRs on cellulolytic activity. The results of Experiment I did not show any significant difference in the degree of Avicel hydrolysis between enzymes incubated with or without LRRs or any difference due to the severity factor and/or botanical origin of the LRRs (Fig. 2a–c). On the contrary, the results of Experiment II showed that the glucose release from Avicel was reduced after preincubation with LRRs (Fig. 2d–f); corroborating that some of the enzymes were adsorbed to the insoluble fraction, leaving reduced activity in the supernatant. The reductions were around 19–28, 30–57 and 31–52% for LRRs from CS, MS and WS, respectively across all time points and severity factors; although there was no significant difference among the LRRs in response to the degree of severity factor (Fig. 2d–f).

In Experiment I, despite the adsorption of enzymes from the commercial cellulolytic enzyme mixture on LRRs (Fig. 1), there was no reduction of enzymatic activity in saccharifying cellulose (Fig. 2a–c). Since the final enzyme dosage in the hydrolysis reaction was low (10 mg protein/g DM cellulose) and on par with that being used in large-scale process [28], it is less likely that the absence of any retardation of the glucose release was due to the excess of unbound enzymes. Furthermore, in Experiment II, the activity of unbound enzymes after adsorption was studied in a scenario as if the binding of the enzymes on lignin were irreversible by performing solid–liquid separation. The results showed that the activity of unbound enzymes alone (with reductions of 19–57% for the different LRRs across all time points) was not enough to degrade the added Avicel to the same rate and extent as that accomplished by the free enzymes that were not adsorbed on LRRs (Fig. 2d–f). This absence of retardation in Experiment I therefore indicated that the LRR-adsorbed cellulose degrading enzymes in the mixture were still active on the added cellulose despite the adsorption. The phenomenon has been shown previously where the enzymes adsorbed on insoluble lignin-rich solids obtained after hydrolysis of dilute acid pretreated corn stover can be recycled by adding fresh substrate to the residue [29]. The finding thus led to two possibilities.

One possibility is that the enzymes were still active despite being bound on lignin [30, 31], likely since the binding occurred without impeding the active site of the enzymes. However, if this was the sole case, a pronounced decrease in cellulose hydrolysis should still be observed since adsorbed or immobilized cellulases were reported to have decreased activity [31, 32]. In contrary, Experiment I showed negligible effect of the presence of LRRs on enzymatic cellulose hydrolysis (Fig. 2a–c). Experiment I has been designed to expand the assessment of enzyme–lignin interaction. On the one hand, the preadsorption of the enzymes on lignin provided more difficulty for the enzymes to degrade the cellulose. On the other hand, the latter introduction of cellulose allowed the enzymes to display whether the binding on lignin is reversible. Therefore, by showing negligible difference in the Avicel hydrolysis (Fig. 2a–c), the results gave strong indication on the reversible binding nature of the enzymes on lignin, i.e. the more likely was the possibility that the adsorbed enzymes desorbed from the LRRs and then re-adsorbed onto Avicel and catalyzed the degradation. This is also supported by the previous findings that cellulases have higher affinity to cellulose or lignocellulosic biomass compared to lignin [16, 33]. The adsorption and desorption kinetics of individual monocomponent cellulases on lignin hence deserve further investigation in order to corroborate this hypothesis.

It is also important to note that the trend of the effect of LRRs on the enzymes was consistent throughout the tested grass biomass (Fig. 2a–c). In a previous study, LRR from hydrothermally pretreated spruce was found to reduce the rate and extent of Avicel hydrolysis by cellulases after incubation at 45 °C [13]. In this work, there was no reduction discernible as a result of the presence of LRRs during reaction at 50 °C (Fig. 2a–c). The difference between the data in this study and the previously reported data on spruce is likely due to botanical origin of the lignin material. Hence this present work, along with other studies using LRRs from hydrothermally pretreated grasses, showed that the LRRs did not reduce the rate and extent of enzymatic cellulose hydrolysis despite some degree of enzyme adsorption on the LRRs [26, 34, 35].

Experiment II nevertheless validated that a portion of the commercial cellulolytic enzyme mixture did get adsorbed onto LRRs and thus was not recovered when the supernatant was transferred, thereby reducing the extent of Avicel hydrolysis (Fig. 2d–f). Since glucose release was compromised in Experiment II, it is plausible that the fraction of the adsorbed enzyme consisted of β-glucosidases (BGLs). Accordingly, BGL was reported previously to have the highest affinity toward lignin from steam-pretreated corn stover compared to other components of Cellic^®^ CTec2 [36]. However, the difference in the reduction of Avicel hydrolysis in Experiment II may be a result of the different affinities of other various enzyme components in the mixture to the different LRRs, as there was no distinct pattern in the overall protein adsorption (Fig. 1). Since the LRRs did not exert nonproductive adsorption and reduction of activity that distinguished them from one another, it can be expected that there were only minor changes in the chemical composition of the lignin.

### Physical and chemical characterization of the lignin-rich residues

A series of physical and chemical characterizations were performed on the LRRs to assess any physicochemical changes in the lignin after HTP at different severity factors and to understand the role of lignin as a physical barrier. Firstly, nuclear magnetic resonance (NMR) spectroscopy was performed mainly to assess the relative abundance of inter-unit linkages in the lignin polymer of the LRRs after pretreatment at different severities. The ^13^C-^1^H HSQC (heteronuclear single quantum coherence) spectra (Additional file 1: Figures S4–S12) revealed that there was a slight decrease of β-*O*-4 linkage in all biomasses with each elevated severity level. The C–C bonds (β-5 and β–β) however, remained relatively stable except for a slight increase of β-5 in MS (Table 2).

**Table 2:** ^13^C-^1^H HSQC contour integration values for inter-unit linkages in the lignin polymer of the lignin-rich residues

Structure	CS			MS			WS		
	3.65	3.83	3.97	3.65	3.83	3.97	3.65	3.83	3.97
G2	1	1	1	1	1	1	1	1	1
β-*O*-4	*	0.46	0.40	0.49	0.44	0.41	0.63	0.57	0.55
β-5	n/a	n/a	n/a	0.08	0.10	0.18	0.08	0.10	0.10
β–β	n/a	n/a	n/a	nd	0.005	nd	0.010	0.010	nd

G2: C_2_-H_2_ correlation peak in guaiacyl subunit was used as reference

n/a: not applicable, structure does not exist

nd: peak too small for accurate determination

* Contour integration was not possible

The reduction of ether β-*O*-4 linkages intensity is expected to occur as a result of increasing pretreatment severity as it is the most susceptible linkage to break during thermochemical treatment [19, 37–39]. However, the cleavage of β-*O*-4 was not significant with only 4–13% signal reduction in the contour integration values across biomass and severity levels (Table 2). In another study, an increase of HTP severity factor (log *R*_0_) from 2.76 (160 °C for 10 min) to 3.65 (190 °C for 10 min) resulted in a 700% drop of the relative abundance of β-*O*-4 linkages in wheat straw lignin [39]. The minimal change in β-*O*-4 cleavage observed in this study therefore can be attributed to the narrower range of the HTP severity factor (log *R*_0_) being used (from 3.65 to 3.97). Stable contour integration values of covalent C–C bonds have also been reported across elevated severity either due to increase of temperature or addition of acid [39]. Overall, the 2D NMR results suggested that the lignin in the LRRs did not undergo significant chemical changes within the tested severity factors.

**Fig. 3:**
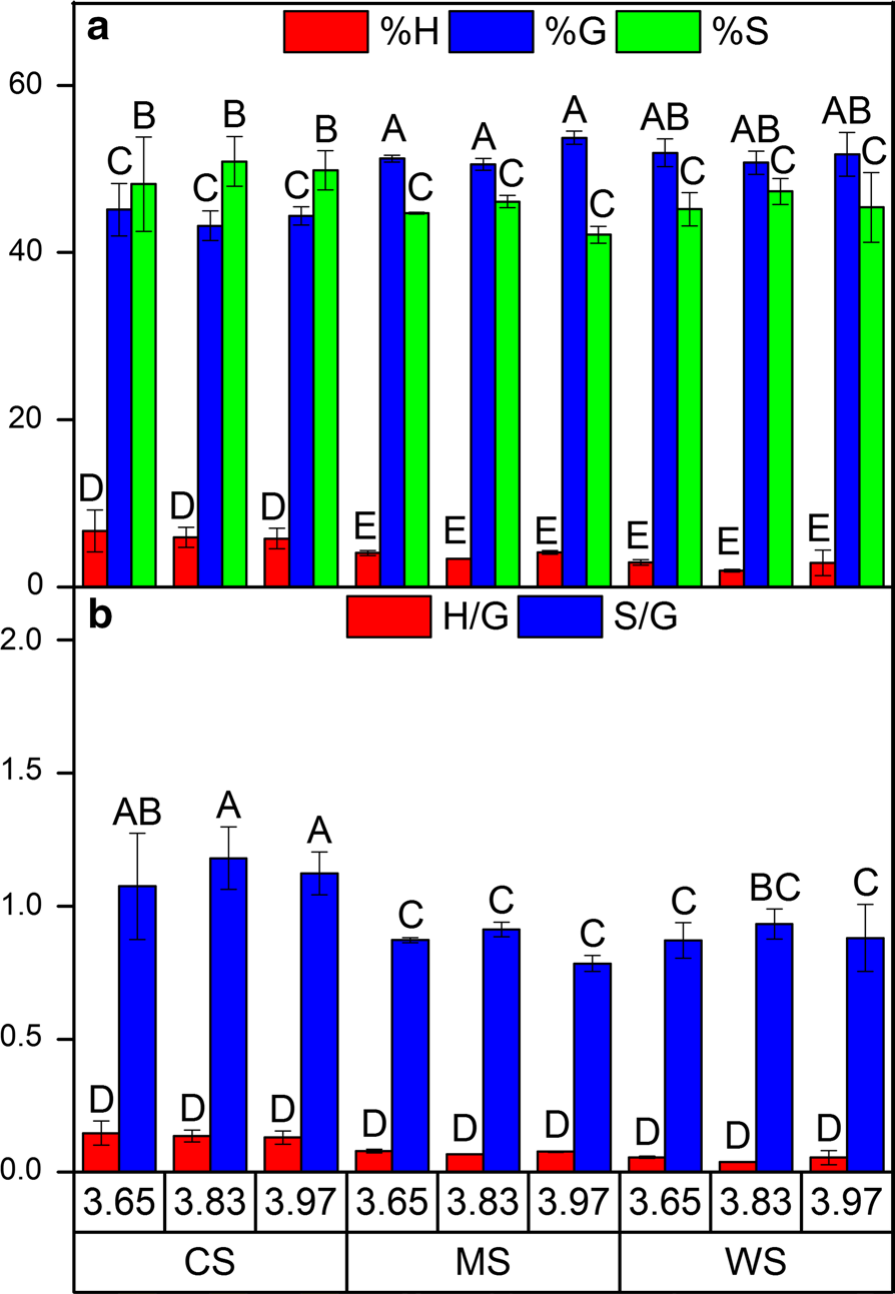
The relative abundance of monolignols, namely *p*-hydroxyphenyl (*H*), guaiacyl (*G*), and syringyl (*S*) units (**a**), and the corresponding monolignols ratio (**b**), based on Py-GC–MS results of the lignin-rich residues (LRRs) from corn stover (CS), *Miscanthus *× *giganteus* stalks (MS), and wheat straw (WS). Data points represent average and standard deviation from two replicates. Different letters (A, B, C, …) indicate significant statistical difference based on ANOVA (*P* ≤ 0.05)

Py-GC–MS analysis was performed in order to assess the composition of the monolignols of the different lignin-rich residues (LRRs) from corn stover (CS), *Miscanthus *× *giganteus* stalks (MS) and wheat straw (WS). The results revealed no differences on the relative monolignols contents after pretreatment with different severities and their corresponding ratios in the case of each biomass (Fig. 3). However, there were differences of relative monolignols contents among biomass feedstocks. LRRs from CS had higher content of syringyl (*S*) and *p*-hydroxyphenyl (*H*) units while lower content of guaiacyl (*G*) unit compared to the other biomasses (Fig. 3a), which resulted in higher *S*/*G* ratio of CS compared to MS and WS (Fig. 3b).

**Fig. 4:**
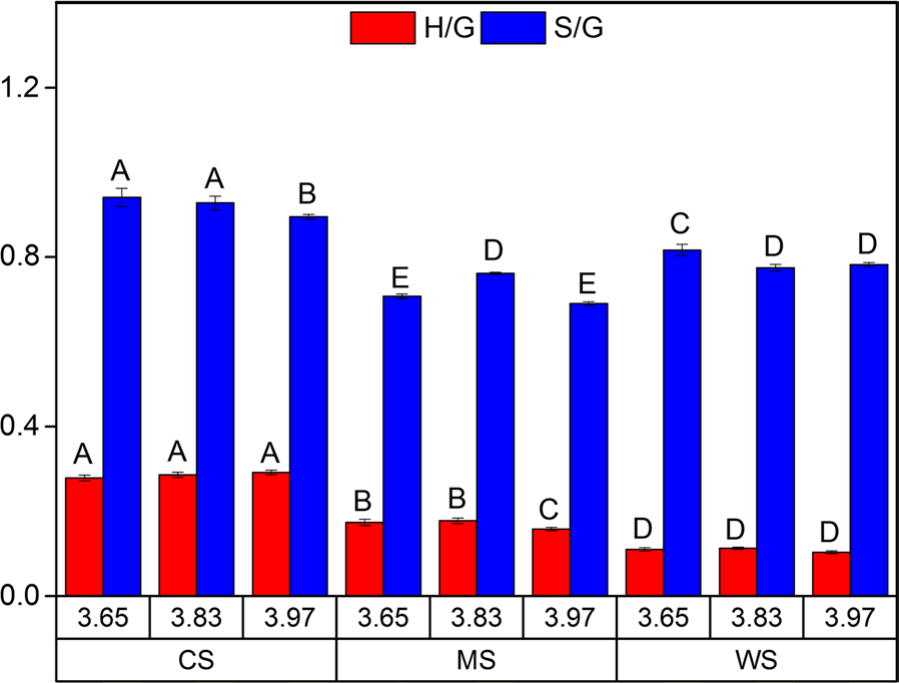
Monolignols ratios (peak area ratio of 835/1508 and 1601/1508 cm^−1^ for *H*/*G* and *S*/*G* ratio, respectively) based on ATR-FTIR spectroscopy results of the lignin-rich residues (LRRs) from corn stover (CS), *Miscanthus *× *giganteus* stalks (MS) and wheat straw (WS) hydrothermally pretreated at different pretreatment severity factors (log *R*_0_). Data points represent average and standard deviation from five replicates. Different letters (A, B, C, …) indicate significant statistical difference based on ANOVA (*P* ≤ 0.05)

Attenuated total reflectance—Fourier Transform Infrared (ATR-FTIR) spectroscopy has also been used to estimate monolignols ratios, although it has been done in different ways in different studies [40–43]. Py-GC–MS and NMR are more commonly used to assess *S*/*G* ratio [38, 44]. In this study, *H*/*G* and *S*/*G* ratios were assessed by calculating the ratio of estimated peak areas of 835/1508 and 1601/1508 cm^−1^, respectively (Additional file 1: Figures S1–S3). The wavenumbers 835, 1508, and 1601 cm^−1^ each corresponds to a signal from *H*, *G*, and *S* units, respectively (Table 3) [40]. The calculated monolignols ratio (Fig. 4) resembled those determined by Py-GC–MS (Fig. 3b) both in terms of number and trend, which confirms that no changes in the monolignols ratio took place as a result of the increased pretreatment severity, but distinct differences were evident due to the inherent differences among the biomass feedstocks. The work indicates that the ATR-FTIR spectroscopy approach can be used as a fast method to estimate monolignols’ ratio of lignin from grasses. In retrospect, the finding also showed that the chemical composition of the lignin on the surface of the LRRs (using ATR-FTIR) was the same as that of the bulk of the LRRs (using Py-GC–MS). This congruence of results between ATR-FTIR and Py-GC–MS on the chemical composition of the lignin corroborated the aforementioned notion that the residual carbohydrates in the LRRs (Table 1) were engulfed by the same lignin which covered the surface of the LRR particles.

**Table 3:** ATR-FTIR assignments of wavenumbers used to measure peak area

Wavenumber (cm^−1^)	Asssignment^a^		Estimated penetration depth^a^ (μm)
835	Lignin	C–H out-of-plane in all position of H and in positions 2 and 6 of *S* units [40]	1.99
895	Holocellulose	Anomeric C-groups, C_1_-H deformation, ring valence vibration (cellulose, wood, holocellulose) [63]	1.85
1419; 1432	Lignin	Aromatic skeletal vibrations combined with C–H in-plane deformation [40]	1.17; 1.16
1508	Lignin	Aromatic skeletal vibrations; *G* > *S* [40, 63]	1.10
1601	Lignin	Aromatic skeletal vibrations plus C=O stretch; *S* > *G* [40, 63]	1.04
1732	Hemicellulose	C=O stretch in unconjugated carbonyl groups of carbohydrate origin (side chain acetylation in mannan, carboxylic acid side chain in xylan and ester groups in lignin–carbohydrate complexes) [40, 63]	0.96

^a^Calculated based on the formula (Eq. 1): $d_{\text{p}} = \frac{\lambda }{{2\pi n_{1} \sqrt {\sin^{2} \theta - \left( {n_{2} /n_{1} } \right)^{2} } }}$ (1) where *d*_p_, *λ*, *θ*, *n*_1_ and *n*_2_ are penetration depth, wavelength, incident angle, ATR crystal refractive index and sample refractive index respectively. The values of *θ* and *n*_1_ are specifically known to be 45° and 2.40 respectively for diamond ATR. The refractive index of biomass samples is estimated to be 1.4 which is a common value for an organic polymer, e.g. in wood cell walls [64]

Other studies assessing biomass resulting from hydrothermal or dilute acid pretreated biomass have found that increasing pretreatment severity results in higher release of *S* units compared to *G* units in GS type lignin, thus reducing the *S*/*G* ratio [19, 45, 46]. However, it has also been reported that the *S*/*G* ratio is unaffected by pretreatment severity level [44, 47]. Apparently, the botanical origin of lignin as well as pretreatment method affect the *S*/*G* ratio. Since cleavage of β-*O*-4 linkages in the LRRs was minimum across the applied severity levels in this study (Table 2), it is conceivable that the *S*/*G* ratio did not change as β-*O*-4 linkage constitutes a significant fraction of linkages with syringyl units [19]. Even though the monolignols content, especially the *S*/*G* ratio of lignin, may be related to biomass recalcitrance, the exact contribution is not clearly defined as conflicting trends across different feedstocks and pretreatment methods have been reported [7].

In this work, the results from Experiment II showed that the LRRs from CS had higher *S*/*G* ratio compared to the others (Figs. 3b and 4) and also gave less reduction of Avicel hydrolysis compared to LRRs from MS and WS (Fig. 2d–f). Accordingly, another study on isolated lignins from hardwood found that there were less adsorption on substrate with higher *S*/*G* ratio [48]. However, this was not apparent from the adsorption experiment since the binding of total protein on LRRs from CS was not significantly lower than others (Fig. 1). Regardless, relative monolignols contents (Figs. 3 and 4) indicated that the chemical composition of the lignin did not change significantly across the tested hydrothermal pretreatment severity levels.

**Fig. 5:**
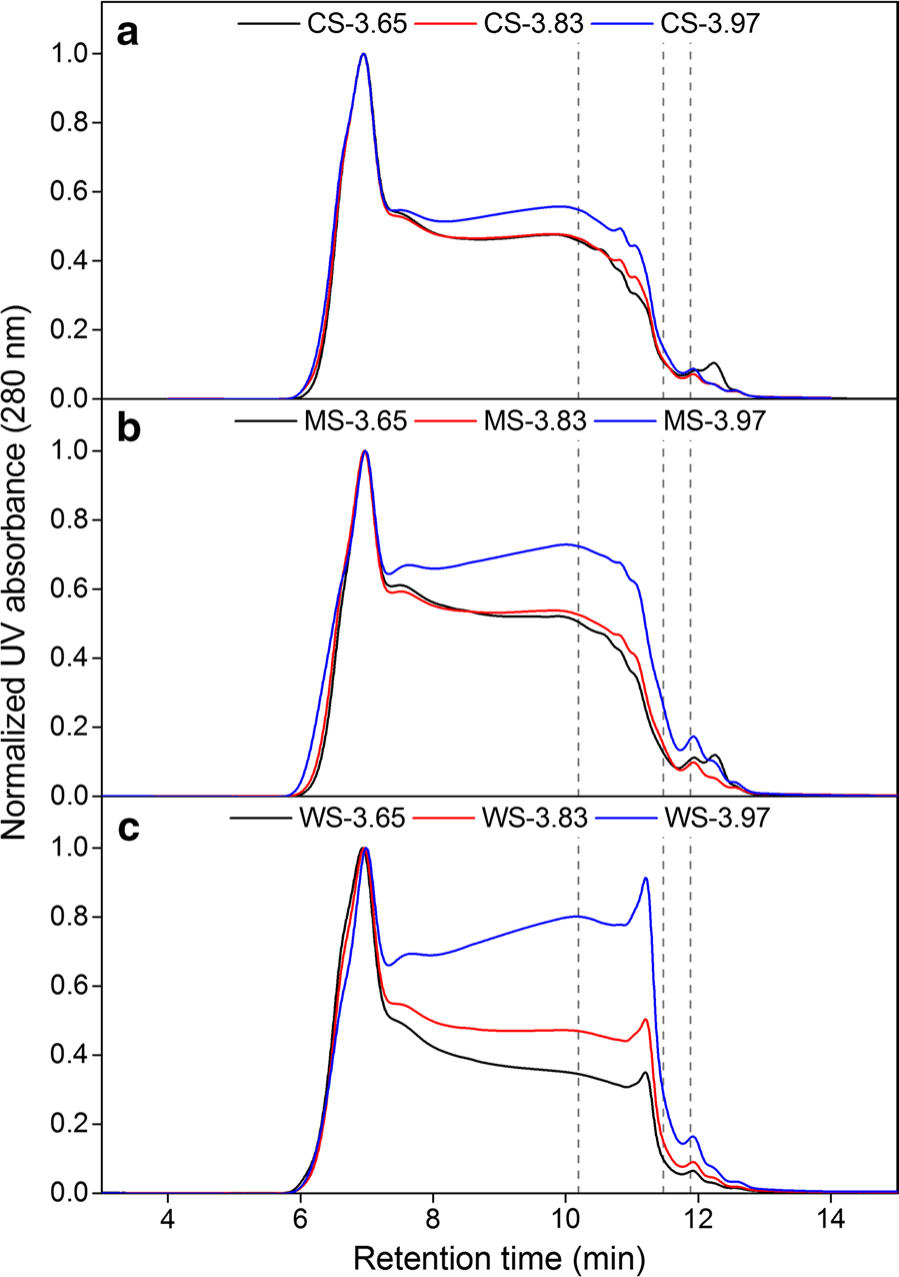
Chromatograms from GPC analysis of lignin-rich residues (LRRs) from corn stover (CS), *Miscanthus *× *giganteus* stalks (MS) and wheat straw (WS) hydrothermally pretreated at different pretreatment severity factors (log *R*_0_). Peaks appearing at higher retention time correspond to fractions with lower molecular weights (*M*_w_). The vertical lines represent standards with *M*_w_ of 1701, 320 and 152 Da appearing at 10.19, 11.47 and 11.88 min, respectively

Gel permeation chromatography (GPC) analysis was performed to assess changes in the physical parameter of lignin, namely molecular weight (*M*_w_) distribution. The absorbance of the compounds at 280 nm was normalized to show the relative changes of *M*_w_ as pretreatment severity was increased. The results of GPC revealed negligible changes in the *M*_w_ fractions of LRRs from CS and MS as the severity increased, except the appearance of low *M*_w_ fractions at the highest severity level tested in this study. However in the case of LRRs from WS, there were substantial increase of fractions with lower *M*_w_ as the severity level was increased (Fig. 5).

During HTP, the cleavage of inter-unit linkages such as β-*O*-4 bonds can occur, which will result in depolymerization of lignin polymer and subsequent decrease of *M*_w_. On the other hand, at increased severity, condensation reactions can also occur due to the formation of new covalent bonds (C–C) which will result in lignin repolymerization and subsequent increase of *M*_w_. Both reactions can occur competitively or either one can dominate [37, 39, 49–51], most likely subject to the botanical origin of the lignin as well as employed pretreatment method and severity factors. The GPC results therefore suggested that there were significantly more depolymerization reaction in the LRRs of WS across severity factors compared to that of CS and MS (Fig. 5).

Based on the 2D NMR results, depolymerization should be likely to have occurred although not to a great extent due to only minor reduction of β-*O*-4 linkage across the tested severity levels in the LRRs (Table 2). The slight increase in the β-5 bond of LRRs from MS (Table 2) can give indication of repolymerization reactions although it can be difficult to confirm. In any case, it is possible that competing repolymerization reactions might have occurred in the lignin polymers of LRRs from CS and MS which resulted in relatively stable distribution of molecular weight fractions across the severity levels. Alternatively, it has been known that guaiacyl (*G*) units tend to start condensation reaction more easily than the syringyl (*S*) units during thermochemical pretreatment and therefore are harder to remove [19, 45, 48, 49, 51]. Based on Py-GC–MS and ATR-FTIR, LRRs of MS and WS had lower *S*/*G* ratio than that of CS (Figs. 3b and 4); meaning that they had more *G* units. However, GPC results showed differently in which lignin depolymerization occurred to a greater extent in the LRRs of WS than others (Fig. 5).

**Fig. 6:**
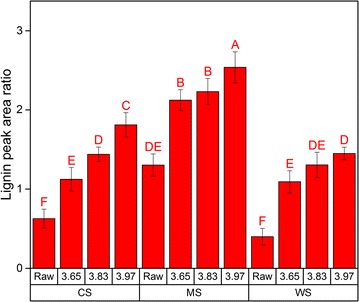
Apparent surface abundance of lignin relative to cellulose (ASA-L/C) of the raw and hydrothermally pretreated corn stover (CS), *Miscanthus giganteus* stalks (MS), and wheat straw (WS) at different severity factors (log *R*_0_) as measured by peak area ratio of 1508/895 cm^−1^ using ATR-FTIR as published in our previous work [24]. Figure was adapted for reprint under Creative Commons Attribution License 4.0. Data points represent average and standard deviation from five replicates. Different letters (A, B, C, …) indicate significant statistical differences based on ANOVA (*P* ≤ 0.05)

Recently, Jensen et al. hypothesized that the presence of tricin, an electron-rich aromatic compound can retard repolymerization reactions [39]. NMR analysis of raw (untreated) biomass revealed more pronounced presence of tricin in WS compared to CS and MS (Additional file 1: Figures S13–S15, Table S2). This can explain the observed reduction of *M*_w_ in the LRRs of WS (Fig. 5), namely due to better prevention of repolymerization reactions and thus the higher extent of lignin depolymerization compared to the LRRs of CS and MS. Accordingly, an effort in gene silencing which reduced the synthesis of tricin in corn revealed that the resulting plant obtained increased recalcitrance due to higher lignin content with covalent interunit linkages [52]. In our previous work, the lignin content of pretreated WS at the three severities was not lower compared to CS and MS (Table 1) [24]. However, our previous finding also pointed that the apparent surface abundance of lignin in the pretreated biomass was thee highest on MS for each corresponding severity factor (Fig. 6), which correlated to its lowest glucose release after enzymatic hydrolysis [24]. Therefore, it is likely that the explanation of the role of lignin in retarding enzymatic cellulose degradation lies in the inherent properties and/or subsequent lignin surface coverage after HTP.

This work showed that when viewing the LRRs as an exaggerated version of extensive cellulose hydrolysis, the residual carbohydrates contents were highest in the LRRs of MS (Table 1). The low extent of hydrolysis of MS correlated to our previous finding, where using ATR-FTIR, we previously showed that MS had the highest initial relative abundance of lignin in the surface prior to pretreatment (Fig. 6) [24]. Following HTP, the apparent surface abundance of lignin in the biomass prior to enzymatic treatment increased with increasing severity factor although it was consistently lower in WS compared to CS and MS for each corresponding severity (Fig. 6) [24]. Extrapolation of the insight from our previous finding to the present study suggests that as extensive cellulose hydrolysis progressed, the advance was retarded earlier in CS and MS which had higher apparent surface abundance of lignin than WS. This lower apparent surface abundance of lignin in pretreated WS (Fig. 6) corresponded to a greater extent of depolymerization of lignin in the LRRs of WS (Fig. 5). In contrast, the lignin in pretreated CS and MS with higher apparent surface abundance (Fig. 6) corresponded to lesser extent of lignin depolymerization (Fig. 5); indicating the possibility that lignin properties can affect its subsequent distribution. Therefore, it can be suggested that the lignin in pretreated CS and MS has become a more potent physical barrier that shielded the carbohydrates after pretreatment than the lignin in pretreated WS. This proposition is supported by the fact that the remaining carbohydrates in the LRRs (Table 1) were not accessible for release by enzymes (Additional file 1: Table S1) and that ATR-FTIR revealed increased presence of lignin in the surface but almost negligible carbohydrates (Additional file 1: Figures S1–S3). All in all, this study along with our previous work [24] and recent works by other groups [53–55] have emphasized the role of lignin as a physical barrier which hinders the accessibility of enzymes to the cellulose in lignocellulosic biomass during extended enzymatic treatment.

## Conclusions

The study showed that enzymes from a commercial cellulolytic mixture adsorbed onto lignin-rich residues (LRRs) isolated from hydrothermally pretreated grass biomass. Although the adsorption reduced the free activity in the supernatant, the performance of the enzymes was not affected by the presence of LRRs. The applied pretreatment severity levels did not significantly affect lignin’s chemical composition, and while there were differences across biomass feedstocks, the differences had no impact on the adsorption of enzymes and their ability to saccharify cellulose. On the other hand, even though there was a positive correlation between the lignin content of the LRRs with severity level and biomass digestibility, the residual carbohydrates were not accessible due to physical obstruction by lignin. We suggest that the lignin surface coverage, plausibly due to its inherent physicochemical and structural properties, determines the degree of retardation of enzymatic cellulose degradation in lignocellulosic biomass feedstocks. Therefore, the role of lignin in impeding enzymatic degradation of cellulose can be defined more as a physical barrier which obstructs the access of enzymes to cellulose rather than acting as an “inhibitor” that promotes the loss of activity through nonproductive adsorption. This points to the need to better understand pretreatment and hydrolysis of biomass particles at the physical level where among others, the migration of lignin can be monitored both within micro- and ultrastructural scales of the plant cell wall. Based on the results obtained in the present study, it is also important to investigate the dynamics of nonproductive binding of cellulases and its monocomponent enzymes on lignin in order to assess if the adsorption of individual enzymes differs with various substrates.

## Methods

### Biomass feedstocks

Corn stover (*Zea mays* subsp. *mays* L.) (CS), *Miscanthus *×* giganteus* stalks (MS), and wheat straw (*Triticum aestivum* L.) (WS) were each hydrothermally pretreated at three severity factors (log *R*_0_): 3.65 (190 °C, 10 min), 3.83 (190 °C, 15 min), and 3.97 (195 °C, 15 min) as described previously [24]. Composition of biomass fiber fraction was determined using strong acid hydrolysis procedure based on the protocol of the National Renewable Energy Laboratory (NREL) [56].

### Isolation of lignin-rich residues (LRRs)

Isolation of the LRRs was performed according to Rahikainen et al. [13] with modifications. Extensive cellulose hydrolysis of the biomass was performed using Cellic^®^ CTec3 (Novozymes, Bagsværd, Denmark) with a dosage of 60 mg protein/g DM biomass in 0.05 M sodium citrate buffer pH 5.0 at 50 °C with 7.5% DM solids loading for 72 h. After every 24 h of hydrolysis, the whole suspension was centrifuged, and then, fresh amount of buffer and enzyme were added as in the original amount. After 72 h, the suspension was sieved using 500-μm mesh and washed thrice using ultrapure water pH 2.50 acidified with HCl, freeze-dried, and then protease treated. The protease treatment of the residue was done using commercial protease from *Bacillus licheniformis* (Sigma-Aldrich, St. Louis, MO, USA) at 37 °C for 24 h at 5% DM solids loading and enzyme loading of 20 mg protein/g DM residue) in 0.5 M NaHCO_3_/Na_2_CO_3_ buffer pH 9.60. After protease treatment, the LRRs were freeze-dried, ground, and used for analyses and experiments. Composition of the LRRs was determined using strong acid hydrolysis procedure [56]. Elemental analysis was performed on the LRRs to confirm the removal of adsorbed proteins using an EA3000 element analyzer with acetanilide as standard (Euro Vector Instruments & Software, Milan, Italy). After protease treatment, the nitrogen content was significantly reduced, indicating that the remaining adsorbed enzymes after extensive hydrolysis had been removed (Additional file 1: Table S3).

### Adsorption experiment

Adsorption experiment was performed using Cellic^®^ CTec3 (Novozymes A/S, Bagsværd, Denmark) with protein loading of 20 mg/g DM. The experiments were performed in triplicates at 1% DM lignin-rich residues in 0.05 M sodium citrate buffer pH 5.0 in 2 ml Protein LoBind^®^ tubes (Eppendorf AG, Hamburg, Germany). The tubes were agitated using a tube revolver (Thermo Fisher Scientific Inc., Waltham, MA, USA) for 2 h at 15 RPM and incubated at 50 °C. After the experiments, liquid fractions were separated by centrifugation and stored frozen prior to analysis. The protein concentration in the liquid fraction was analyzed using ninhydrin method with bovine serum albumin (BSA) as standard [57, 58].

### Effect of lignin-rich residues (LRRs) on the hydrolysis of cellulose

The effect of the isolated LRRs on the enzymatic hydrolysis of cellulose was assessed in two experiments. In Experiment I, 1 ml of 0.2 mg protein/ml Cellic^®^ CTec3 (Novozymes A/S, Bagsværd, Denmark) was incubated with 1% DM LRRs as in the previous adsorption experiment study for 2 h at 50 °C. Then 0.5 ml suspension of 6% DM Avicel PH-101 (Sigma-Aldrich, St. Louis, MO, USA) was added into the mixture which resulted in final total solids concentration of 2.67% DM. The final enzyme dosage being used in Experiment I therefore was 6.7 mg protein/g DM total solids (LRR and Avicel) or equivalent to 10 mg protein/g DM cellulose (Avicel). As a control, the same amount of enzyme was incubated without LRRs. In Experiment II, 1 ml of 0.2 mg protein/ml Cellic^®^ CTec3 (Novozymes A/S, Bagsværd, Denmark) was also incubated with 1% DM LRRs as in the previous adsorption experiment study for 2 h at 50 °C. The suspension was then centrifuged, and 0.75 ml of the supernatant was mixed with 0.375 ml of 6% DM Avicel PH-101 (Sigma-Aldrich, St. Louis, MO, USA) suspension which resulted in final solids concentration of 2% DM. As a control, the same amount of liquid was taken from the same amount of enzyme that was incubated without the LRRs. In both experiments, the hydrolysis of added Avicel was performed in ThermoMixer Comfort (Eppendorf AG, Hamburg, Germany) at 50 °C and agitation of 1250 RPM with samples being taken after 1, 6 and 24 h. Samples for each time points were boiled for 10 min, centrifuged, and the supernatant was analyzed for glucose using Dionex ICS-5000 system (DionexCorp, Sunnyvale, CA, USA) as explained previously [24]. Both experiments were performed in duplicate and enzyme and substrate blanks were used for correction.

### Pyrolysis-gas chromatography–mass spectrometry (Py-GC–MS)

The lignin-rich residues were pyrolyzed in duplicates according to Jensen et al. [59] with modifications. The samples were prepared by transferring about 100–200 µg to a pyrolysis tube. Pyrolysis was performed under a He flow of 100 ml/min at 500 °C (calibrated as sample received temperature). The pyrolysis temperature was held for 20 s by the PYRO pyrolysis unit (GERSTEL, Mülheim an der Ruhr, Germany). The transfer line was held at 320 °C and pyrolysates were carried onto the chromatographic column with a 100:1 split in the inlet held at 300 °C. The pyrolysates were separated and detected using 7890B GC and 5977A MSD series GC–MS (Agilent, St. Clara, CA, USA) equipped with an VF-5 ms (60 m, 0.25 mm, 0.25 µm) (Agilent, St. Clara, CA, USA) column. All compounds used for calculating monolignol ratios were identified by standards or published mass spectra [60]. The compounds were grouped according to methoxylation into *H*, *G* or *S* (Additional file 1: Table S4). Monolignol ratios were calculated as the peak area of the specific monolignol in proportion to the total peak area of the three monolignols.

### Gel permeation chromatography (GPC)

The molecular weight (*M*_w_) distribution of aromatic compounds in the LRR samples was determined by gel permeation chromatography (GPC) using a procedure adapted from a previous study [59]. Separation of compounds was performed on a PolarSil column (300 × 8 mm, 5 µm, 100 Å) (PSS Polymer Standards Service, Mainz, Germany) at 70 °C in a 9:1 (v/v) dimethyl sulfoxide/water eluent with 0.05 M LiBr. The LRR samples were dissolved in the eluent at concentrations of 2 g/l with sonication and overnight mixing. Detection was performed using UV–Vis detector at 280 nm. Tannic acid (1701 Da), β-*O*-4 dimer (320 Da) and vanillin (152 Da) were used as standards to approximate the molecular weight distribution in the chromatograms.

## 2D Nuclear magnetic resonance (NMR)

The lignin-rich residues were prepared in DMSO-d_6_/pyridine-d_5_ for whole plant cell wall characterization and were analyzed through heteronuclear single quantum coherence (HSQC) experiments according to the established protocol [61] as reported previously [24].

### Attenuated total reflectance-Fourier transform infrared (ATR-FTIR) spectroscopy

ATR-FTIR measurements were performed on the lignin-rich residues with five technical replicates using a Nicolet 6700 FT-IR, Pike Technologies GladiATR diamond spectrometer (Thermo Scientific, Waltham, MA, USA) as described previously [24]. The IR spectra (Additional file 1: Figures S1–S3) were normalized using Standard Normal Variate [62]. The peaks included are listed in Table 3. Monolignols ratios of syringyl (*S*), guaiacyl (*G*) and *p*-hydroxyphenyl (*H*) units of lignin, namely *H*/*G* and *S*/*G* ratios, were assessed by calculating the ratio of estimated peak areas of 835/1508 and 1601/1508 cm^−1^ respectively.

### Statistical analysis

One-way analysis of variance (ANOVA) was performed using JMP 13 (SAS Institute Inc., Cary, NC, USA) with post hoc analysis using Tukey–Kramer’s Honestly Significant Difference (HSD) test at *P* ≤ 0.05. Connecting letters were used to report the significant statistical difference among the mean values where different letters indicate that the compared mean values are significantly different. For example, values with the letters “A,” “B,” and “C” are significantly different from one another, whereas those with the letter “A” are not significantly different.

## Additional file

**Additional file 1: Table S1.** Glucose and xylose release after hydrolysis of lignin-rich residues using Cellic^®^ CTec3. **Figures S1**–**S3.** ATR-FTIR spectra of lignin-rich residues (LRRs) isolated from hydrothermally pretreated (log *R*_*0*_ = 3.65, 3.83 and 3.97) corn stover (CS), *Miscanthus* × *giganteus* stalks (MS) and wheat straw (WS) and the corresponding spectra of the fiber fraction of pretreated biomass. **Figures S4**–**S12.** ^13^C-^1^H HSQC (heteronuclear single quantum coherence) spectra of lignin-rich residues (LRRs) isolated from hydrothermally pretreated (log *R*_*0*_ = 3.65, 3.83 and 3.97) corn stover (CS), *Miscanthus* × *giganteus* stalks (MS) and wheat straw (WS). **Figures S13**–**S15.** ^13^C-^1^H HSQC (heteronuclear single quantum coherence) spectra of raw (untreated) corn stover (CS), *Miscanthus* × *giganteus* stalks (MS) and wheat straw (WS). **Table S2.** ^13^C-^1^H HSQC contour integration values for tricin in the raw (untreated) biomass feedstocks. **Table S3.** Nitrogen content of the pretreated biomass feedstocks and their corresponding lignin-rich residues (LRRs) after hydrolysis and protease treatment. **Table S4.** Py-GC-MS relative peak areas (%) of compounds used for calculation of monolignol ratios of the lignin-rich residues.

## References

[1] Larsen J, Haven MØ, Thirup L (2012). Inbicon makes lignocellulosic ethanol a commercial reality. Biomass Bioenerg.

[2] Yang B, Tao L, Wyman CE (2018). Strengths, challenges, and opportunities for hydrothermal pretreatment in lignocellulosic biorefineries. Biofuels Bioprod Biorefining.

[3] Ruiz HA, Rodríguez-Jasso RM, Fernandes BD, Vicente AA, Teixeira JA (2013). Hydrothermal processing, as an alternative for upgrading agriculture residues and marine biomass according to the biorefinery concept: a review. Renew Sustain Energy Rev.

[4] Mansfield SD, Mooney C, Saddler JN (1999). Substrate and enzyme characteristics that limit cellulose hydrolysis. Biotechnol Prog.

[5] Himmel ME, Ding S-Y, Johnson DK, Adney WS, Nimlos MR, Brady JW, Foust TD (2007). Biomass recalcitrance: engineering plants and enzymes for biofuels production. Science.

[6] Jørgensen H, Kristensen JB, Felby C (2007). Enzymatic conversion of lignocellulose into fermentable sugars: challenges and opportunities. Biofuels Bioprod Biorefining.

[7] Li M, Pu Y, Ragauskas AJ (2016). Current understanding of the correlation of lignin structure with biomass recalcitrance. Front Chem.

[8] Sipponen MH, Rahikainen J, Leskinen T, Pihlajaniemi V, Mattinen M-L, Lange H, Crestini C, Österberg M (2017). Structural changes of lignin in biorefinery pretreatments and consequences to enzyme-lignin interactions—OPEN ACCESS. Nord Pulp Pap Res J.

[9] Saini JK, Patel AK, Adsul M, Singhania RR (2016). Cellulase adsorption on lignin: a roadblock for economic hydrolysis of biomass. Renew Energy.

[10] Sewalt VJH, Glasser WG, Beauchemin KA (1997). Lignin impact on fiber degradation. 3. Reversal of inhibition of enzymatic hydrolysis by chemical modification of lignin and by additives. J Agric Food Chem.

[11] Berlin A, Balakshin M, Gilkes N, Kadla J, Maximenko V, Kubo S, Saddler J (2006). Inhibition of cellulase, xylanase and beta-glucosidase activities by softwood lignin preparations. J Biotechnol.

[12] Tu M, Pan X, Saddler JN (2009). Adsorption of cellulase on cellulolytic enzyme lignin from lodgepole pine. J Agric Food Chem.

[13] Rahikainen J, Mikander S, Marjamaa K, Tamminen T, Lappas A, Viikari L, Kruus K (2011). Inhibition of enzymatic hydrolysis by residual lignins from softwood - study of enzyme binding and inactivation on lignin-rich surface. Biotechnol Bioeng.

[14] Lai C, Tu M, Shi Z, Zheng K, Olmos LG, Yu S (2014). Contrasting effects of hardwood and softwood organosolv lignins on enzymatic hydrolysis of lignocellulose. Bioresour Technol.

[15] Ko JK, Ximenes E, Kim Y, Ladisch MR (2015). Adsorption of enzyme onto lignins of liquid hot water pretreated hardwoods. Biotechnol Bioeng.

[16] Palonen H, Tjerneld F, Zacchi G, Tenkanen M (2004). Adsorption of *Trichoderma reesei* CBH I and EG II and their catalytic domains on steam pretreated softwood and isolated lignin. J Biotechnol.

[17] Börjesson J, Engqvist M, Sipos B, Tjerneld F (2007). Effect of poly(ethylene glycol) on enzymatic hydrolysis and adsorption of cellulase enzymes to pretreated lignocellulose. Enzyme Microb Technol.

[18] Kellock M, Rahikainen J, Marjamaa K, Kruus K (2017). Lignin-derived inhibition of monocomponent cellulases and a xylanase in the hydrolysis of lignocellulosics. Bioresour Technol.

[19] Trajano HL, Engle NL, Foston M, Ragauskas AJ, Tschaplinski TJ, Wyman CE (2013). The fate of lignin during hydrothermal pretreatment. Biotechnol Biofuels.

[20] Yelle DJ, Kaparaju P, Hunt CG, Hirth K, Kim H, Ralph J, Felby C (2013). Two-dimensional NMR evidence for cleavage of lignin and xylan substituents in wheat straw through hydrothermal pretreatment and enzymatic hydrolysis. Bioenerg Res.

[21] Donohoe BS, Decker SR, Tucker MP, Himmel ME, Vinzant TB (2008). Visualizing lignin coalescence and migration through maize cell walls following thermochemical pretreatment. Biotechnol Bioeng.

[22] Kristensen JB, Thygesen LG, Felby C, Jørgensen H, Elder T (2008). Cell-wall structural changes in wheat straw pretreated for bioethanol production. Biotechnol Biofuels.

[23] Zeng Y, Himmel ME, Ding SY (2017). Visualizing chemical functionality in plant cell walls. Biotechnol Biofuels.

[24] Djajadi DT, Hansen AR, Jensen A, Thygesen LG, Pinelo M, Meyer AS, Jørgensen H (2017). Surface properties correlate to the digestibility of hydrothermally pretreated lignocellulosic Poaceae biomass feedstocks. Biotechnol Biofuels.

[25] Nakagame S, Chandra RP, Kadla JF, Saddler JN (2011). The isolation, characterization and effect of lignin isolated from steam pretreated Douglas-fir on the enzymatic hydrolysis of cellulose. Bioresour Technol.

[26] Lu X, Zheng X, Li X, Zhao J (2016). Adsorption and mechanism of cellulase enzymes onto lignin isolated from corn stover pretreated with liquid hot water. Biotechnol Biofuels.

[27] Ooshima H, Burns DS, Converse AO (1990). Adsorption of cellulase from *Trichoderma reesei* on cellulose and lignacious residue in wood pretreated by dilute sulfuric acid with explosive decompression. Biotechnol Bioeng.

[28] Larsen J, Øtergaard Petersen M, Thirup L, Li HW, Iversen FK (2008). The IBUS process—lignocellulosic bioethanol close to a commercial reality. Chem Eng Technol.

[29] Weiss N, Börjesson J, Pedersen LS, Meyer AS (2013). Enzymatic lignocellulose hydrolysis: improved cellulase productivity by insoluble solids recycling. Biotechnol Biofuels.

[30] Rodrigues AC, Leitão AF, Moreira S, Felby C, Gama M (2012). Recycling of cellulases in lignocellulosic hydrolysates using alkaline elution. Bioresour Technol.

[31] Lu X, Wang C, Li X, Zhao J, Zhao X (2017). Studying nonproductive adsorption ability and binding approach of cellobiohydrolase to lignin during bioconversion of lignocellulose. Energy Fuels.

[32] Tébéka IRM, Silva AGL, Petri DFS (2009). Hydrolytic activity of free and immobilized cellulase. Langmuir.

[33] Li Y, Sun Z, Ge X, Zhang J (2016). Effects of lignin and surfactant on adsorption and hydrolysis of cellulases on cellulose. Biotechnol Biofuels.

[34] Nakagame S, Chandra RP, Saddler JN (2010). The effect of isolated lignins, obtained from a range of pretreated lignocellulosic substrates, on enzymatic hydrolysis. Biotechnol Bioeng.

[35] Barsberg S, Selig MJ, Felby C (2013). Impact of lignins isolated from pretreated lignocelluloses on enzymatic cellulose saccharification. Biotechnol Lett.

[36] Yarbrough JM, Mittal A, Mansfield E, Taylor LE, Hobdey SE, Sammond DW, Bomble YJ, Crowley MF, Decker SR, Himmel ME, Vinzant TB (2015). New perspective on glycoside hydrolase binding to lignin from pretreated corn stover. Biotechnol Biofuels.

[37] Li J, Henriksson G, Gellerstedt G (2007). Lignin depolymerization/repolymerization and its critical role for delignification of aspen wood by steam explosion. Bioresour Technol.

[38] del Río JC, Rencoret J, Prinsen P, Martinéz ÁT, Ralph J, Gutiérrez A (2012). Structural characterization of wheat straw lignin as revealed by analytical pyrolysis, 2D-NMR, and reductive cleavage methods. J Agric Food Chem.

[39] Jensen A, Cabrera Y, Hsieh C-W, Nielsen J, Ralph J, Felby C (2017). 2D NMR characterization of wheat straw residual lignin after dilute acid pretreatment with different severities. Holzforschung.

[40] Faix O (1991). Classification of lignins from different botanical origins by FT-IR spectroscopy. Holzforschung.

[41] Santos JI, Martín-sampedro R, Fillat Ú, Oliva JM, Negro MJ, Ballesteros M, Eugenio ME, Ibarra D (2015). Evaluating lignin-rich residues from biochemical ethanol production of wheat straw and olive tree pruning by FTIR and 2D-NMR. Int J Polym Sci.

[42] Huang Y, Wang L, Chao Y, Nawawi DS, Akiyama T, Yokoyama T, Matsumoto Y (2012). Analysis of lignin aromatic structure in wood based on the IR spectrum. J Wood Chem Technol.

[43] Derkacheva OY (2013). Estimation of aromatic structure contents in hardwood lignins from IR absorption spectra. J Appl Spectrosc.

[44] Tana T, Zhang Z, Moghaddam L, Rackemann DW, Rencoret J, Gutiérrez A, del Río JC, Doherty WOS (2016). Structural changes of sugar cane bagasse lignin during cellulosic ethanol production process. ACS Sustain Chem Eng.

[45] Wayman M (1979). Chua MGS. Characterization of autohydrolysis aspen (*P. trenieuloides*) lignins. Part 2. Alkaline nitrobenzene oxidation studies of extracted autohydrolysis lignin. Can J Chem.

[46] Li C, Knierim B, Manisseri C, Arora R, Scheller HV, Auer M, Vogel KP, Simmons BA, Singh S (2010). Comparison of dilute acid and ionic liquid pretreatment of switchgrass: biomass recalcitrance, delignification and enzymatic saccharification. Bioresour Technol.

[47] Li M, Cao S, Meng X, Studer M, Wyman CE, Ragauskas AJ, Pu Y (2017). The effect of liquid hot water pretreatment on the chemical—structural alteration and the reduced recalcitrance in poplar. Biotechnol Biofuels.

[48] Yu Z, Gwak K-S, Treasure T, Jameel H, Chang H, Park S (2014). Effect of lignin chemistry on the enzymatic hydrolysis of woody biomass. Chemsuschem.

[49] Samuel R, Pu Y, Raman B, Ragauskas AJ (2010). Structural characterization and comparison of switchgrass ball-milled lignin before and after dilute acid pretreatment. Appl Biochem Biotechnol.

[50] Pu Y, Hu F, Huang F, Davison BH, Ragauskas AJ (2013). Assessing the molecular structure basis for biomass recalcitrance during dilute acid and hydrothermal pretreatments. Biotechnol Biofuels.

[51] Pielhop T, Larrazábal GO, Studer MH, Brethauer S, Seidel C-M, Rudolf von Rohr P (2015). Lignin repolymerisation in spruce autohydrolysis pretreatment increases cellulase deactivation. Green Chem R Soc Chem.

[52] Eloy NB, Voorend W, Lan W, de Saleme MLS, Cesarino I, Vanholme R, Smith RA, Goeminne G, Pallidis A, Morreel K, Nicomedes J, Ralph J, Boerjan W (2017). Silencing chalcone synthase in maize impedes the incorporation of tricin into lignin and increases lignin content. Plant Physiol.

[53] Ju X, Engelhard M, Zhang X (2013). An advanced understanding of the specific effects of xylan and surface lignin contents on enzymatic hydrolysis of lignocellulosic biomass. Bioresour Technol.

[54] Wallace J, Brienzo M, García-Aparicio MP, Görgens JF (2016). Lignin enrichment and enzyme deactivation as the root cause of enzymatic hydrolysis slowdown of steam pretreated sugarcane bagasse. N Biotechnol.

[55] Dumitrache A, Tolbert A, Natzke J, Brown SD, Davison BH, Ragauskas AJ (2017). Cellulose and lignin colocalization at the plant cell wall surface limits microbial hydrolysis of Populus biomass. Green Chem R Soc Chem.

[56] Sluiter A, Hames B, Ruiz R, Scarlata C, Sluiter J, Templeton D, Crocker D (2008). Determination of structural carbohydrates and lignin in biomass.

[57] Starcher B (2001). A ninhydrin-based assay to quantitate the total protein content of tissue samples. Anal Biochem.

[58] Tu M, Chandra RP, Saddler JN (2007). Recycling cellulases during the hydrolysis of steam exploded and ethanol pretreated lodgepole pine. Biotechnol Prog.

[59] Jensen MM, Madsen RB, Becker J, Iversen BB, Glasius M (2017). Products of hydrothermal treatment of lignin and the importance of ortho-directed repolymerization reactions. J Anal Appl Pyrolysis..

[60] Ralph J, Hatfield RD (1991). Pyrolysis-GC–MS characterization of forage materials. J Agric Food Chem.

[61] Mansfield SD, Kim H, Lu F, Ralph J (2012). Whole plant cell wall characterization using solution-state 2D NMR. Nat Protoc.

[62] Barnes RJ, Dhanoa MS, Lister SJ (1989). Standard normal variate transformation and de-trending of near-infrared diffuse reflectance spectra. Appl Spectrosc.

[63] Schwanninger M, Rodrigues JC, Pereira H, Hinterstoisser B (2004). Effects of short-time vibratory ball milling on the shape of FT-IR spectra of wood and cellulose. Vib Spectrosc.

[64] Baas P (1975). Interference microscopic studies on wood plastic and cell wall-liquid interactions in beech. J Microsc.

