# Supplementary material for: Lignin from hydrothermally pretreated grass biomass retards enzymatic cellulose degradation by acting as a physical barrier rather than by inducing nonproductive adsorption of enzymes
Source: Biotechnol Biofuels. 2018 Apr 2;11:85. doi: 10.1186/s13068-018-1085-0 (PMC5880018; doi:10.1186/s13068-018-1085-0)
Supplement: Supplementary file 1 — Additional file 1: Table S1. Glucose and xylose release after hydrolysis of lignin-rich residues using Cellic® CTec3. Figures S1–S3. ATR-FTIR spectra of lignin-rich residues (LRRs) isolated from hydrothermally pretreated (log R0 = 3.65, 3.83 and 3.97) corn stover (CS), Miscanthus × giganteus stalks (MS) and wheat straw (WS) and the corresponding spectra of the fiber fraction of pretreated biomass. Figures S4–S12. 13C-1H HSQC (heteronuclear single quantum coherence) spectra of lignin-rich residues (LRRs) isolated from hydrothermally pretreated (log R0 = 3.65, 3.83 and 3.97) corn stover (CS), Miscanthus × giganteus stalks (MS) and wheat straw (WS). Figures S13–S15. 13C-1H HSQC (heteronuclear single quantum coherence) spectra of raw (untreated) corn stover (CS), Miscanthus × giganteus stalks (MS) and wheat straw (WS). Table S2. 13C-1H HSQC contour integration values for tricin in the raw (untreated) biomass feedstocks. Table S3. Nitrogen content of the pretreated biomass feedstocks and their corresponding lignin-rich residues (LRRs) after hydrolysis and protease treatment. Table S4. Py-GC-MS relative peak areas (%) of compounds used for calculation of monolignol ratios of the lignin-rich residues. [file 13068_2018_1085_MOESM1_ESM.docx]

Supplementary information for:

Lignin from hydrothermally pretreated grass biomass retards enzymatic cellulose degradation by acting as a physical barrier rather than by inducing non-productive adsorption of enzymes

Demi T. Djajadi^1^, Mads M. Jensen^2^, Marlene Oliveira^1^, Anders Jensen^3^, Lisbeth G. Thygesen^3^, Manuel Pinelo^1^, Marianne Glasius^2^, Henning Jørgensen^1,4^, Anne S. Meyer^1*^

Affiliations:

1: Department of Chemical and Biochemical Engineering, Technical University of Denmark, Søltofts Plads Building 229, 2800 Kongens Lyngby, Denmark

2: Department of Chemistry, Aarhus University, Langelandsgade 140, 8000 Aarhus C, Denmark

3: Department of Geosciences and Natural Resource Management, University of Copenhagen, Rolighedsvej 23, 1958 Frederiksberg C, Denmark

4: Present address: Department of Plant and Environmental Sciences, University of Copenhagen, Thorvaldsensvej 40, 1871 Frederiksberg C, Denmark

*Corresponding author: Anne S. Meyer ([am@kt.dtu.dk](mailto:am@kt.dtu.dk))

# Table S1 Glucose and xylose release after hydrolysis of lignin-rich residues using Cellic® CTec3

| **Biomass – log *R_0_*** | **Glucose released (mg/l)** | **% of theoretical maximum glucose release** | **Xylose released (mg/l)** | **% of theoretical maximum xylose release** |
| --- | --- | --- | --- | --- |
| **CS – 3.65** | 134.3 ± 11.9 | 5.8 ± 0.5 | 51.6 ± 3.5 | 8.3 ± 0.6 |
| **CS – 3.83** | 69.3 ± 1.7 | 4.3 ± 0.1 | 19.4 ± 1.8 | 5.3 ± 0.5 |
| **CS – 3.97** | 41.5 ± 4.4 | 5.2 ± 0.5 | 3.9 ± 1.4 | 2.1 ± 0.8 |
| **MS – 3.65** | 81.8 ± 3.4 | 2.2 ± 0.1 | 35.6 ± 1.0 | 5.5 ± 0.2 |
| **MS – 3.83** | 50.3 ± 5.5 | 2.4 ± 0.3 | 13.8 ± 0.5 | 4.1 ± 0.1 |
| **MS – 3.97** | 41.5 ± 3.2 | 3.2 ± 0.2 | nd | nd |
| **WS – 3.65** | 48.9 ± 7.1 | 3.2 ± 0.5 | 25.2 ± 0.5 | 6.6 ± 0.1 |
| **WS – 3.83** | 22.2 ± 3.7 | 2.5 ± 0.4 | 7.7 ± 1.5 | 3.4 ± 0.7 |
| **WS – 3.97** | 15.5 ± 0.2 | 2.7 ± 0.0 | nd | nd |

nd: the values were not detectable since they were below the detection limit of the HPLC system

Enzymatic hydrolysis of 1% DM LRRs was performed at 50°C, pH 5.0 using Cellic® CTec3 dosage of 10 mg protein/g DM for 24 h

# Fig. S1 ATR-FTIR spectra of lignin-rich residues (LRRs) isolated from corn stover (CS) hydrothermally pretreated at different pretreatment severity factors (log *R_0_*) and the corresponding spectra of the fiber fraction of pretreated biomass. Different lines of same colors represent five replicates of the same samples. Vertical lines mark the wave numbers 835, 895, 1419, 1432, 1512, 1601 and 1732 cm^-1^.

# Fig. S2 ATR-FTIR spectra of lignin-rich residues (LRRs) isolated from *Miscanthus* × *giganteus* stalks (MS) hydrothermally pretreated at different pretreatment severity factors (log *R_0_*) and the corresponding spectra of the fiber fraction of pretreated biomass. Different lines of same colors represent five replicates of the same samples. Vertical lines mark the wave numbers 835, 895, 1419, 1432, 1512, 1601 and 1732 cm^-1^.

# Fig. S3 ATR-FTIR spectra of lignin-rich residues (LRRs) isolated from wheat straw (WS) hydrothermally pretreated at different pretreatment severity factors (log *R_0_*) and the corresponding spectra of the fiber fraction of pretreated biomass. Different lines of same colors represent five replicates of the same samples. Vertical lines mark the wave numbers 835, 895, 1419, 1432, 1512, 1601 and 1732 cm^-1^.


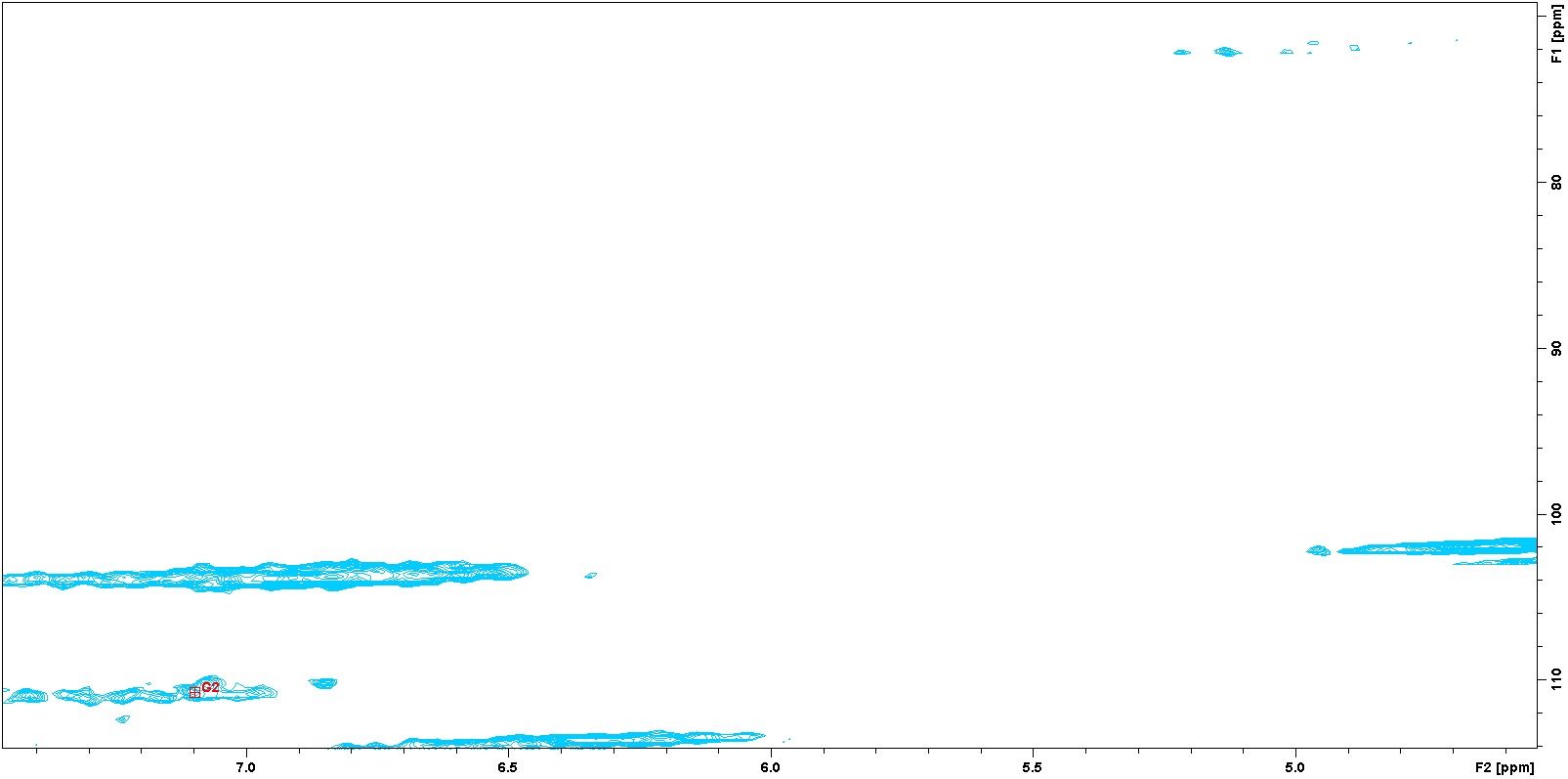


# Fig. S4 ^13^C-^1^H HSQC (heteronuclear single quantum coherence) spectra of lignin-rich residue (LRR) isolated from corn stover (CS) hydrothermally pretreated at log *R_0_* = 3.65 displaying the absence of peaks corresponding to inter-unit linkages relative to the G2 peak (C_2_-H_2_ correlation peak in guaiacyl subunit).


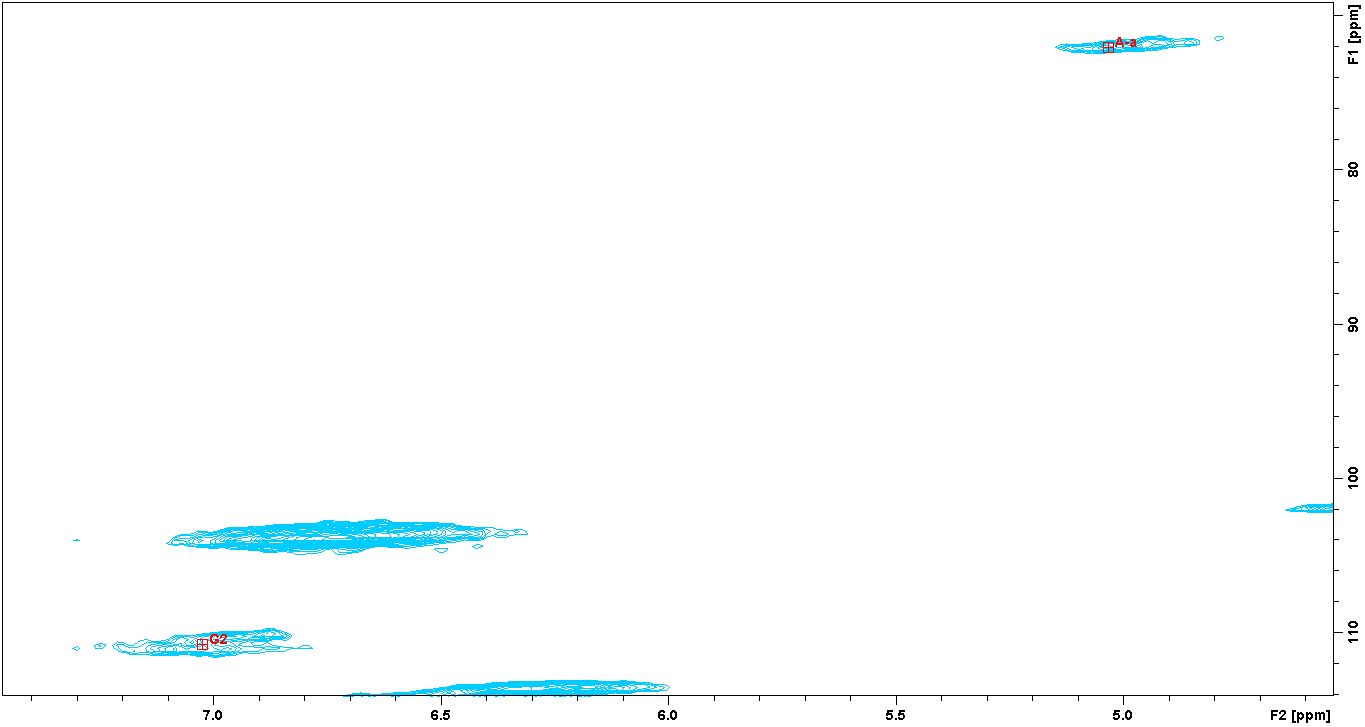


# Fig. S5 ^13^C-^1^H HSQC (heteronuclear single quantum coherence) spectra of lignin-rich residue (LRR) isolated from corn stover (CS) hydrothermally pretreated at log *R_0_* = 3.83 displaying the peak corresponding to β-O-4 linkage (A-a) relative to the G2 peak (C_2_-H_2_ correlation peak in guaiacyl subunit).


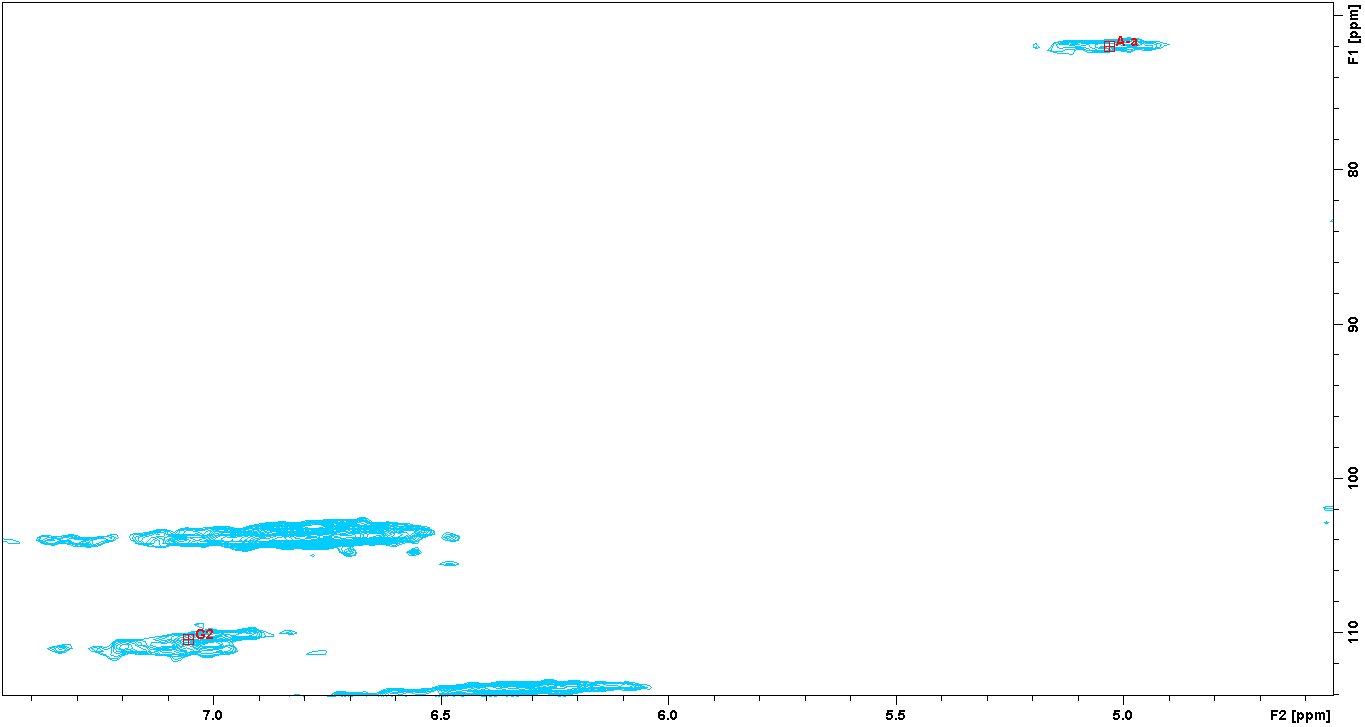


# Fig. S6 ^13^C-^1^H HSQC (heteronuclear single quantum coherence) spectra of lignin-rich residue (LRR) isolated from corn stover (CS) hydrothermally pretreated at log *R_0_* = 3.97 displaying the peak corresponding to β-O-4 linkage (A-a) relative to the G2 peak (C_2_-H_2_ correlation peak in guaiacyl subunit).


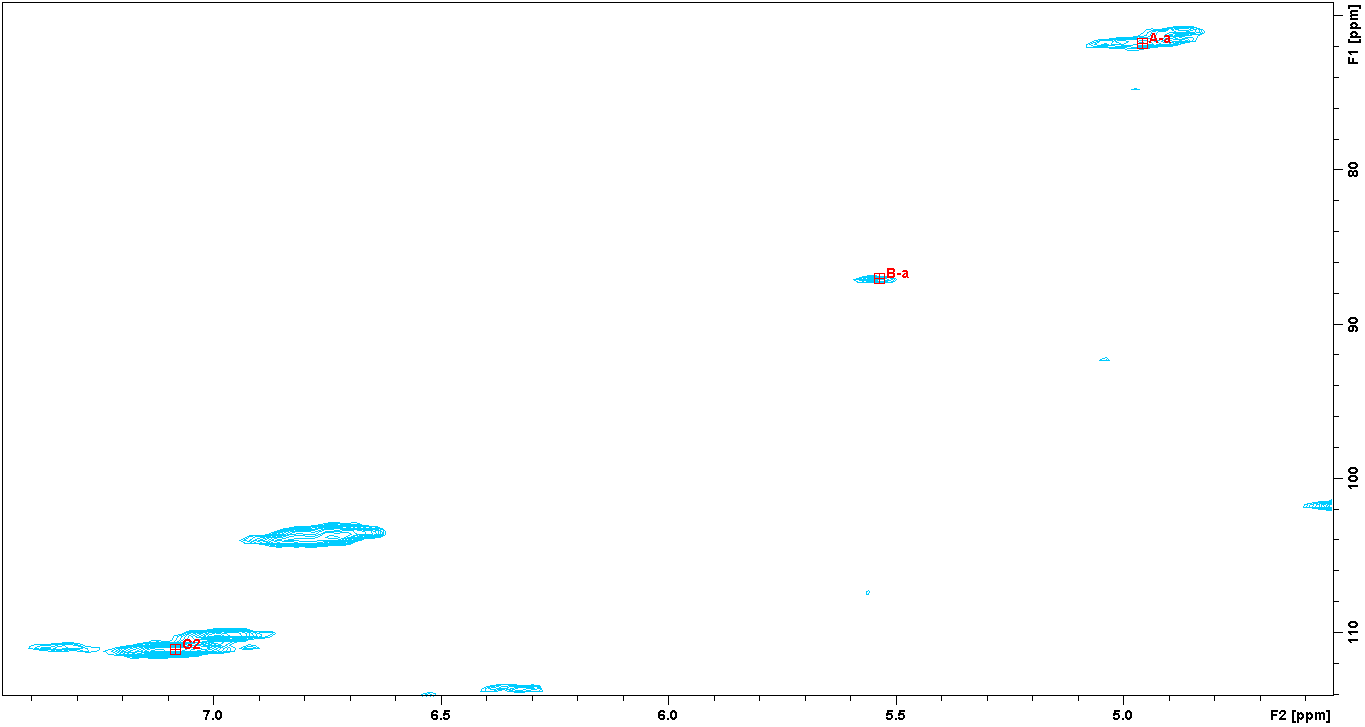


# Fig. S7 ^13^C-^1^H HSQC (heteronuclear single quantum coherence) spectra of lignin-rich residue (LRR) isolated from *Miscanthus* × *giganteus* stalks (MS) hydrothermally pretreated at log *R_0_* = 3.65 displaying the peaks corresponding to inter-unit linkages β-O-4 (A-a) and β-5 (B-a) relative to the G2 peak (C_2_-H_2_ correlation peak in guaiacyl subunit).


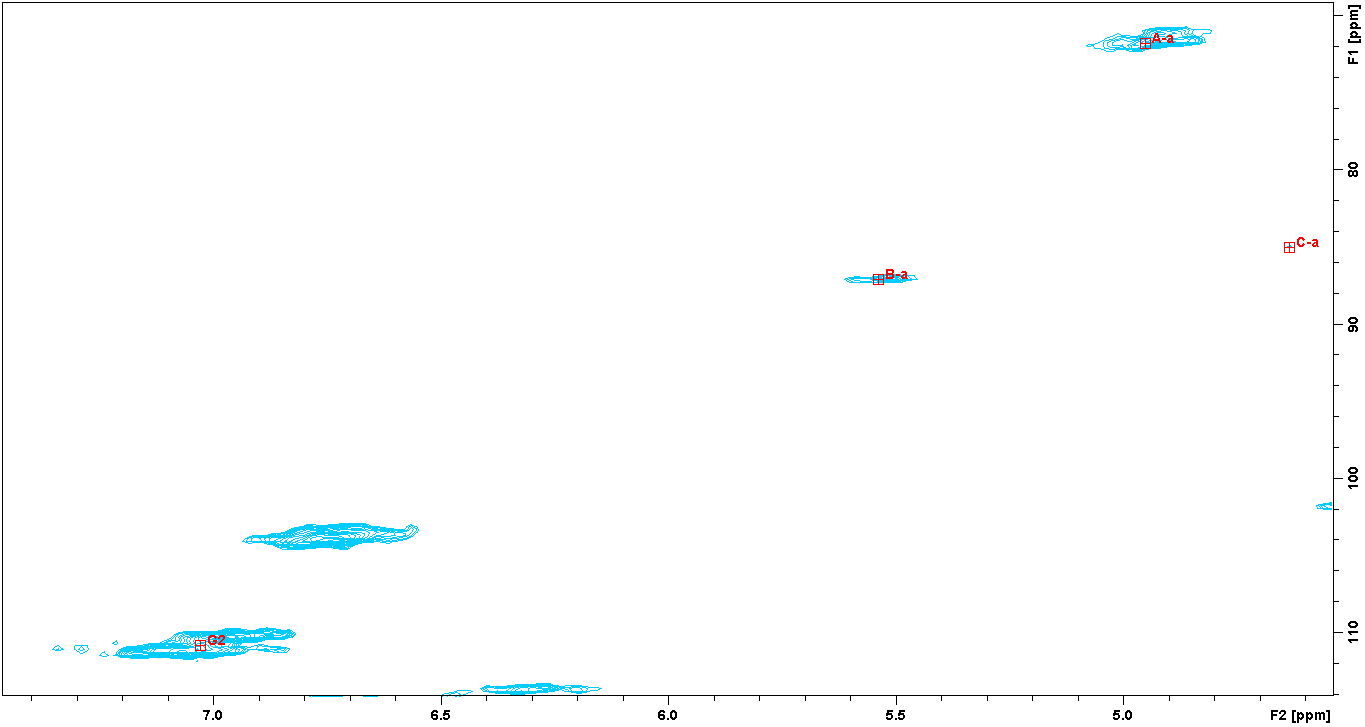


# Fig. S8 ^13^C-^1^H HSQC (heteronuclear single quantum coherence) spectra of lignin-rich residue (LRR) isolated from *Miscanthus* × *giganteus* stalks (MS) hydrothermally pretreated at log *R_0_* = 3.83 displaying the peaks corresponding to inter-unit linkages β-O-4 (A-a), β-5 (B-a) and β-β (C-a) relative to the G2 peak (C_2_-H_2_ correlation peak in guaiacyl subunit).


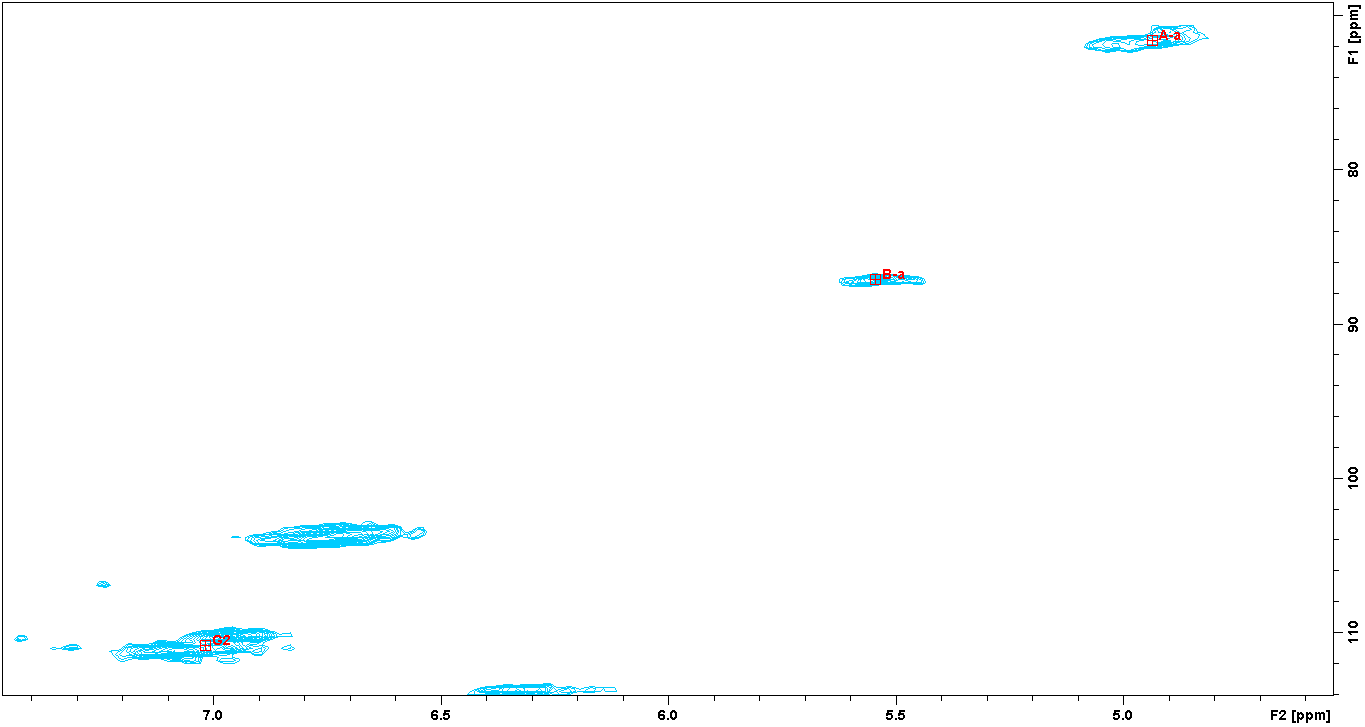


# Fig. S9 ^13^C-^1^H HSQC (heteronuclear single quantum coherence) spectra of lignin-rich residue (LRR) isolated from *Miscanthus* × *giganteus* stalks (MS) hydrothermally pretreated at log *R_0_* = 3.97 displaying the peaks corresponding to inter-unit linkages β-O-4 (A-a) and β-5 (B-a) relative to the G2 peak (C_2_-H_2_ correlation peak in guaiacyl subunit).


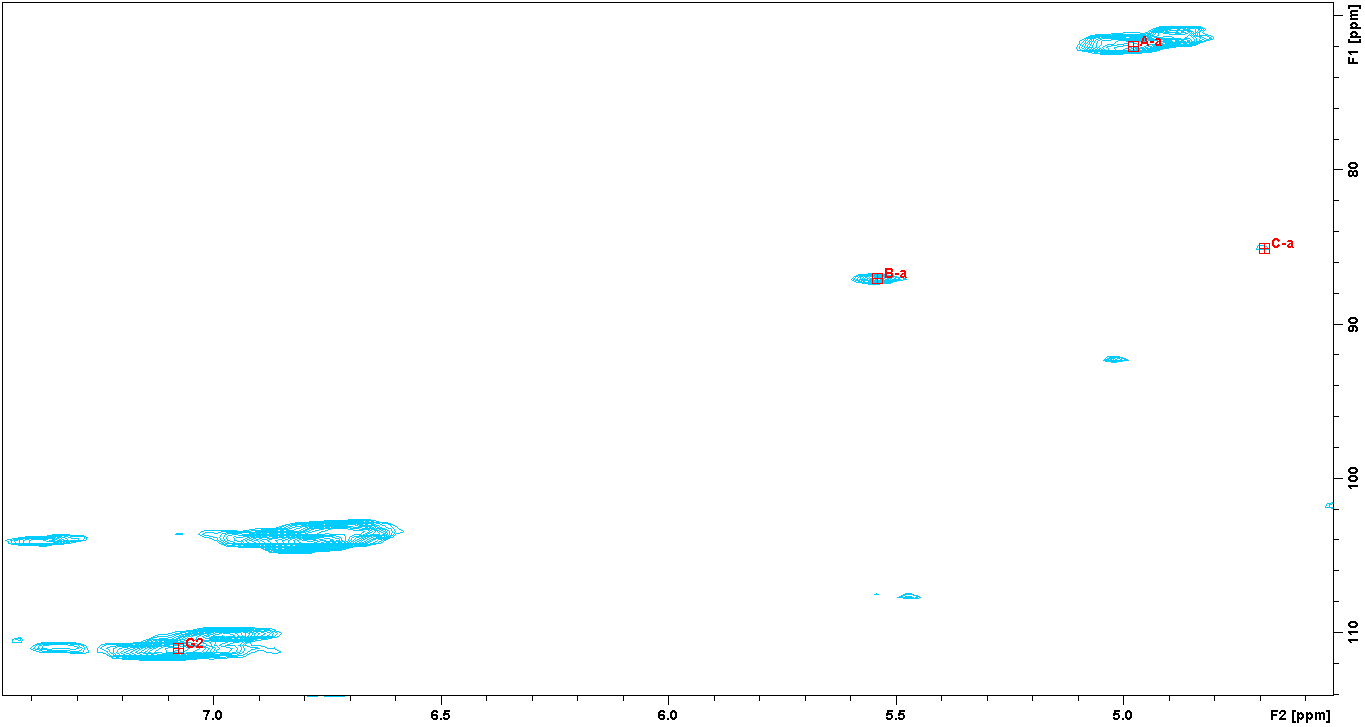


# Fig. S10 ^13^C-^1^H HSQC (heteronuclear single quantum coherence) spectra of lignin-rich residue (LRR) isolated from wheat straw (WS) hydrothermally pretreated at log *R_0_* = 3.65 displaying the peaks corresponding to inter-unit linkages β-O-4 (A-a), β-5 (B-a) and β-β (C-a) relative to the G2 peak (C_2_-H_2_ correlation peak in guaiacyl subunit).


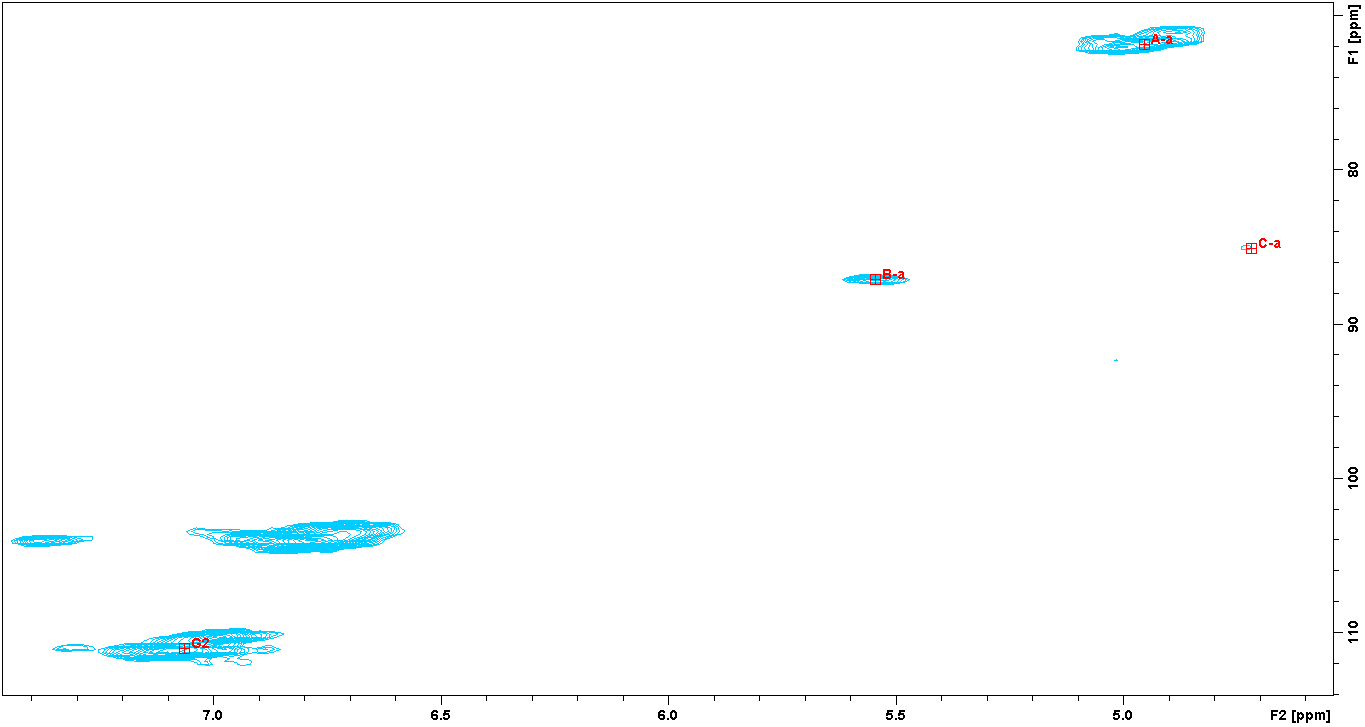


# Fig. S11 ^13^C-^1^H HSQC (heteronuclear single quantum coherence) spectra of lignin-rich residue (LRR) isolated from wheat straw (WS) hydrothermally pretreated at log *R_0_* = 3.83 displaying the peaks corresponding to inter-unit linkages β-O-4 (A-a), β-5 (B-a) and β-β (C-a) relative to the G2 peak (C_2_-H_2_ correlation peak in guaiacyl subunit).


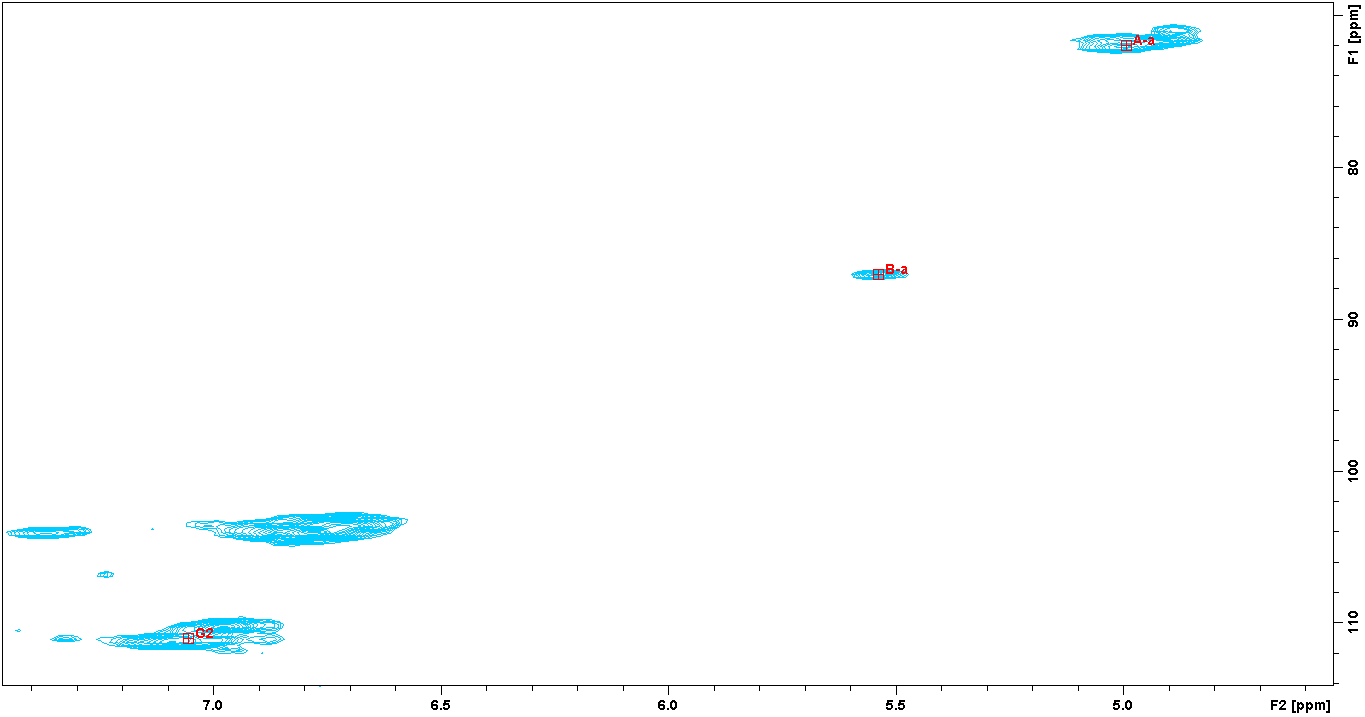


# Fig. S12 ^13^C-^1^H HSQC (heteronuclear single quantum coherence) spectra of lignin-rich residue (LRR) isolated from wheat straw (WS) hydrothermally pretreated at log *R_0_* = 3.97 displaying the peaks corresponding to inter-unit linkages β-O-4 (A-a) and β-5 (B-a) relative to the G2 peak (C_2_-H_2_ correlation peak in guaiacyl subunit).


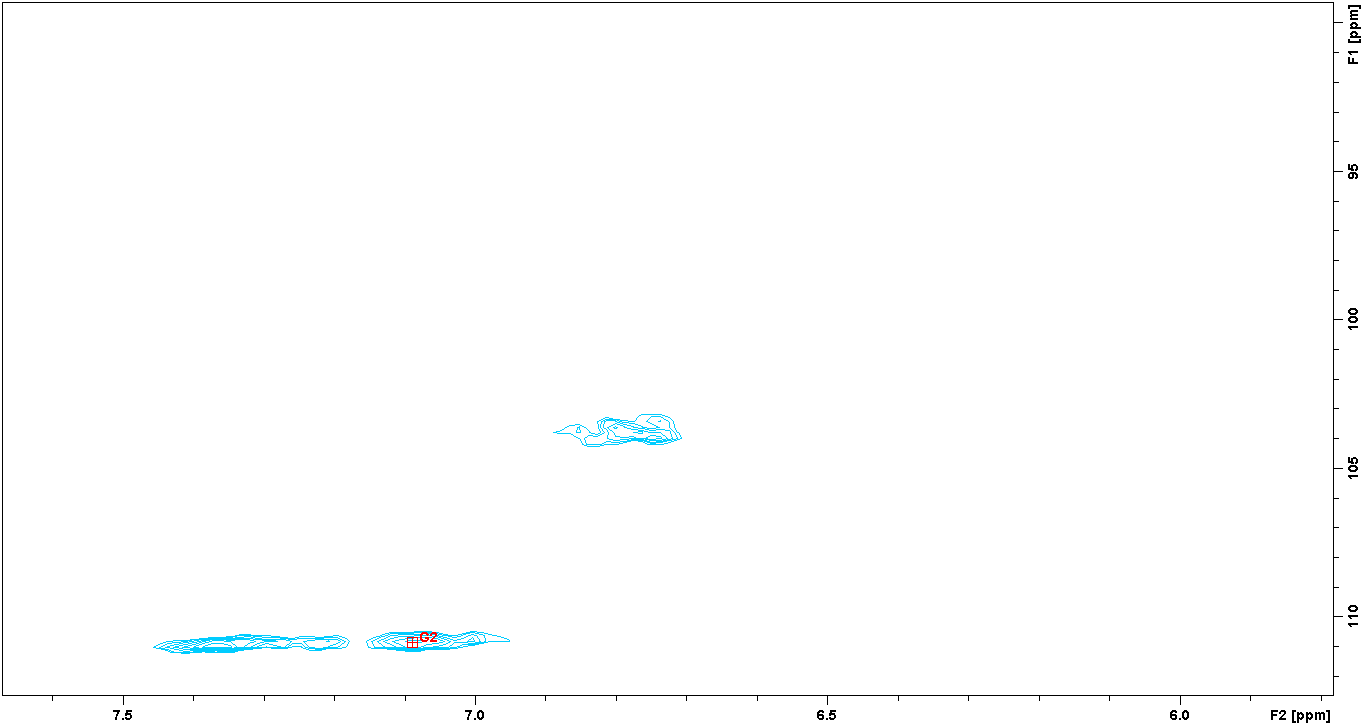


# Fig. S13 ^13^C-^1^H HSQC (heteronuclear single quantum coherence) spectra of raw (untreated) corn stover (CS) displaying the absence of tricin peaks relative to the G2 peak (C_2_-H_2_ correlation peak in guaiacyl subunit).


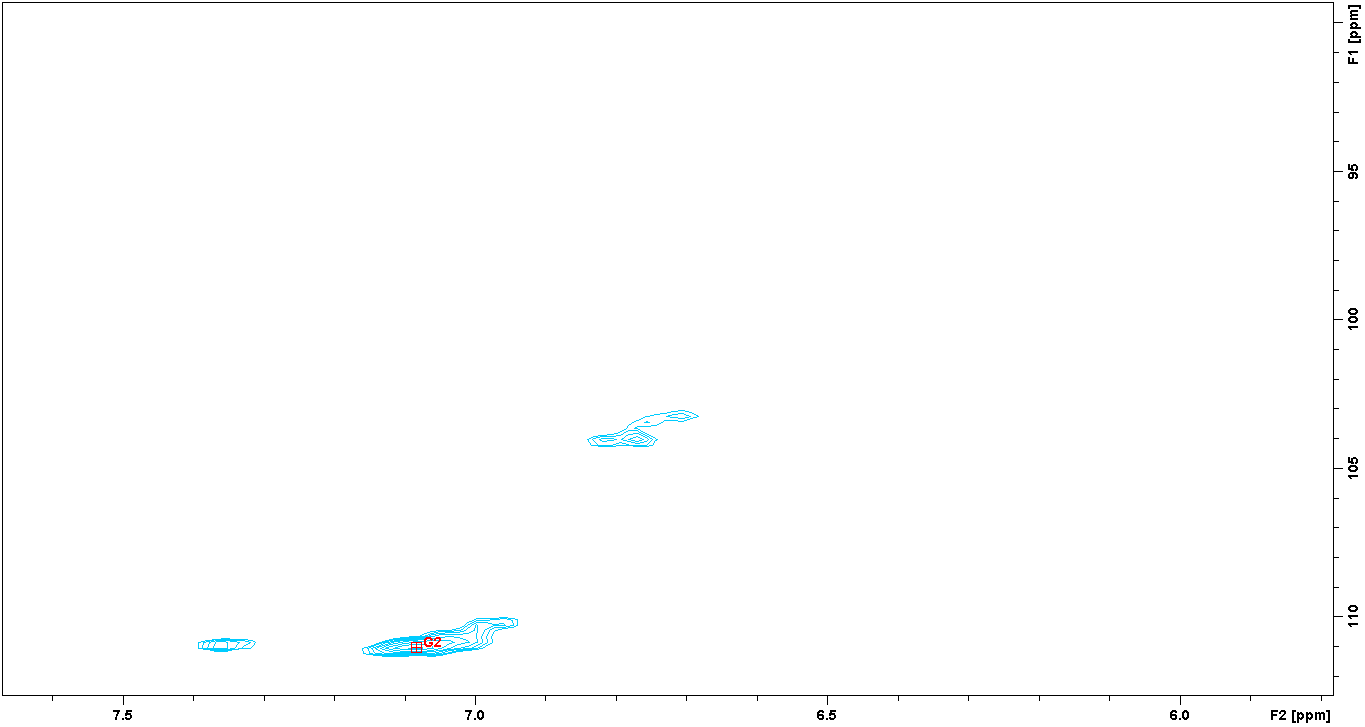


# Fig. S14 ^13^C-^1^H HSQC (heteronuclear single quantum coherence) spectra of raw (untreated) *Miscanthus* × *giganteus* stalks (MS) displaying the absence of tricin peaks relative to the G2 peak (C_2_-H_2_ correlation peak in guaiacyl subunit).


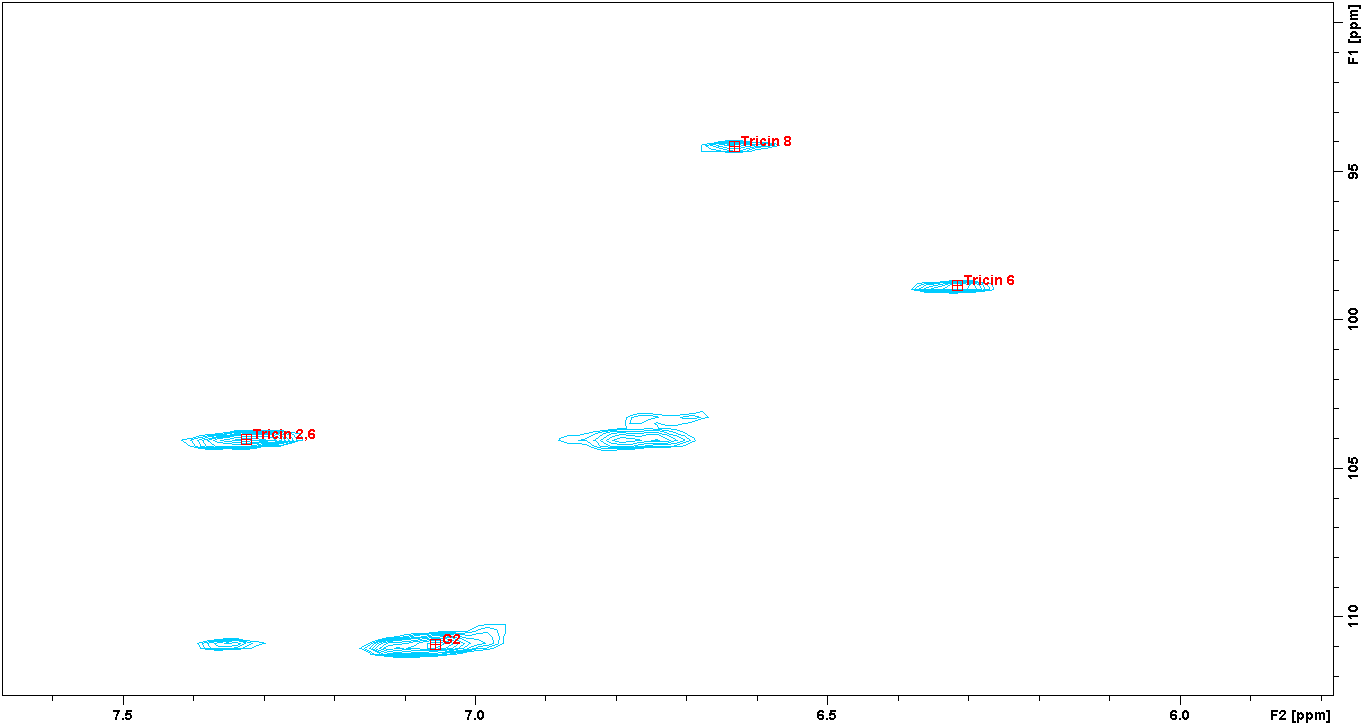


# Fig. S15 ^13^C-^1^H HSQC (heteronuclear single quantum coherence) spectra of raw (untreated) wheat straw (WS) displaying the tricin peaks (Tricin 2,6; Tricin 6; Tricin 8) relative to the reference G2 peak (C_2_-H_2_ correlation peak in guaiacyl subunit).

# Table S2 ^13^C-^1^H HSQC contour integration values for tricin in the raw (untreated) biomass feedstocks

| **Structure** | **CS** | **MS** | **WS** |
| --- | --- | --- | --- |
| **G2** | 1 | 1 | 1 |
| **Tricin 2,6** | nd | nd | 0.48 |
| **Tricin 6** | nd | nd | 0.24 |
| **Tricin 8** | nd | nd | 0.23 |

CS: corn stover; MS: *Miscanthus* × *giganteus* stalks; WS: wheat straw

G2: C_2_-H_2_ correlation peak in guaiacyl subunit was used as reference

nd: peak too small for accurate determination

# Table S3 Nitrogen content of the pretreated biomass feedstocks and their corresponding lignin-rich residues (LRRs) after hydrolysis and protease treatment

| **%N (w/w)** | | | |
| --- | --- | --- | --- |
| **Biomass – log *R_0_*** | **Pretreated biomass feedstocks** | **LRRs after hydrolysis** | **LRRs after protease treatment** |
| **CS-3.65** | 1.08 ± 0.11 | 2.64 ± 0.04 | 0.94 ± 0.08 |
| **CS-3.83** | 1.06 ± 0.19 | nd | 0.81 ± 0.09 |
| **CS-3.97** | 0.84 ± 0.10 | nd | 0.99 ± 0.08 |
| **MS-3.65** | 1.22 ± 0.13 | 2.08 ± 0.24 | 0.59 ± 0.00 |
| **MS-3.83** | 0.97 ± 0.04 | nd | 0.73 ± 0.01 |
| **MS-3.97** | 0.96 ± 0.05 | nd | 0.93 ± 0.01 |
| **WS-3.65** | 1.16 ± 0.18 | 2.47 ± 0.27 | 0.81 ± 0.14 |
| **WS-3.83** | 0.81 ± 0.07 | nd | 0.82 ± 0.01 |
| **WS-3.97** | 0.84 ± 0.04 | nd | 0.88 ± 0.04 |

CS: corn stover; MS: *Miscanthus* × *giganteus* stalks; WS: wheat straw

Results are average and standard deviation of triplicate measurements

nd: not determined

# Table S4 Py-GC-MS relative peak areas (%) of compounds used for calculation of monolignol ratios of the lignin-rich residues

| Compound | M^+^ (*m*/*z*) | Compound class* | CS-3.65 | CS-3.83 | CS-3.97 | MS-3.65 | MS-3.83 | MS-3.97 | WS-3.65 | WS-3.83 | WS-3.97 |
| --- | --- | --- | --- | --- | --- | --- | --- | --- | --- | --- | --- |
| 4-Ethylphenol | 122 | H | 7 | 6 | 6 | 4 | 3 | 4 | 3 | 2 | 3 |
| Guaiacol | 124 | G | 29 | 28 | 30 | 31 | 32 | 35 | 31 | 32 | 33 |
| 4-Ethylguaiacol | 152 | G | 7 | 7 | 7 | 7 | 6 | 7 | 9 | 8 | 8 |
| Vanillin | 152 | G | 3 | 3 | 3 | 4 | 3 | 3 | 4 | 3 | 3 |
| *trans*-Isoeugenol | 164 | G | 6 | 5 | 5 | 10 | 9 | 8 | 8 | 8 | 7 |
| Syringol | 154 | S | 30 | 32 | 32 | 28 | 30 | 27 | 25 | 27 | 25 |
| 4-Ethylsyringol | 182 | S | 4 | 5 | 4 | 4 | 4 | 4 | 4 | 4 | 4 |
| Syringaldehyde | 182 | S | 2 | 2 | 2 | 2 | 2 | 2 | 2 | 2 | 2 |
| *trans* 4-propenylsyringol | 194 | S | 7 | 7 | 6 | 8 | 8 | 7 | 6 | 6 | 5 |
| Acetosyringone | 196 | S | 5 | 6 | 6 | 3 | 3 | 3 | 8 | 8 | 8 |
| Monolignol sum |  |  | 100 | 100 | 100 | 100 | 100 | 100 | 100 | 100 | 100 |

*H: *p*-hydroxyphenyl, G: guaiacyl, S: syringyl

Compounds used for calculating monolignols ratio were based on previous publication [1]

Datapoints represent averages of duplicate measurement

Reference

1. Ralph J, Hatfield RD. Pyrolysis-GC-MS characterization of forage materials. J. Agric. Food Chem. 1991;39:1426–37.
